# Cancer cell spheroids are a better screen for the photodynamic efficiency of glycosylated photosensitizers

**DOI:** 10.1371/journal.pone.0177737

**Published:** 2017-05-17

**Authors:** Patrícia M. R. Pereira, Naxhije Berisha, N. V. S. Dinesh K. Bhupathiraju, Rosa Fernandes, João P. C. Tomé, Charles Michael Drain

**Affiliations:** 1 QOPNA, Department of Chemistry, University of Aveiro, Aveiro, Portugal; 2 IBILI, Faculty of Medicine, University of Coimbra, Coimbra, Portugal; 3 Department of Chemistry, Hunter College of the City University of New York, New York, New York, United States of America; 4 CNC.IBILI, Faculty of Medicine, University of Coimbra, Coimbra, Portugal; 5 CQE, Instituto Superior Técnico, Universidade de Lisboa, Lisboa, Portugal; 6 Graduate Center of the City University of New York, New York, New York, United States of America; 7 The Rockefeller University, New York, New York, United States of America; Instituto Nacional de Cardiologia, MEXICO

## Abstract

Photodynamic Therapy (PDT) relies on the use of non-toxic photosensitizers that are locally and selectively activated by light to induce cell death or apoptosis through reactive oxygen species generation. The conjugation of porphyrinoids with sugars that target cancer is increasingly viewed as an effective way to increase the selectivity of PDT. To date, *in vitro* PDT efficacy is mostly screened using two-dimensional monolayer cultures. Compared to monolayer cultures, three-dimensional spheroid cultures have unique spatial distributions of nutrients, metabolites, oxygen and signalling molecules; therefore better mimic *in vivo* conditions. We obtained 0.05 mm^3^ spheroids with four different human tumor cell lines (HCT-116, MCF-7, UM-UC-3 and HeLa) with appropriate sizes for screening PDT agents. We observed that detachment from monolayer culture and growth as tumor spheroids was accompanied by changes in glucose metabolism, endogenous ROS levels, galectin-1 and glucose transporter GLUT1 protein levels. We compared the phototoxic responses of a porphyrin conjugated with four glucose molecules (PorGlu_4_) in monolayer and spheroid cultures. The uptake and phototoxicity of PorGlu_4_ is highly dependent on the monolayer *versus* spheroid model used and on the different levels of GLUT1 protein expressed by these *in vitro* platforms. This study demonstrates that HCT-116, MCF-7, UM-UC-3 and HeLa spheroids afford a more rational platform for the screening of new glycosylated-photosensitizers compared to monolayer cultures of these cancer cells.

## Introduction

Photodynamic therapy (PDT) is growing as a non-invasive alternative to chemotherapy and ionizing radiation to treat cancer [1–5]. PDT triggers cell death in cancer cells by formation of reactive oxygen species (ROS) generated by a photosensitizer (PS) when irradiated by light [1–9]. PDT can offer double selectivity *via* selective light irradiation such that areas not irradiated are unaffected [3], and the PS can be chemically modified to target specific tissues or environments, such as specific ligands on cancer cells or the low pH surrounding the milieu of a tumor [6]. Porphyrins are aromatic heterocyclic organic dyes that absorb intensely in the red region of the visible spectrum that is able to furthest penetrate tissue and skin. This makes porphyrins practical for applications in biochemical tracking, diagnostic imaging, and therapies such as PDT. Since the porphyrin core is not soluble in aqueous solutions, it is substituted with solubilizing groups and/or targeting moieties such as polyethyleneglycol or carbohydrates such as glucose and galactose [6, 10–12]. Our prior work demonstrated that glycosylated porphyrins with four glucose units (PorGlu_4_) can be rapidly and efficiently synthesized and targets various cancer cell types [13, 14]. The glycosylated dye was shown to be selectively taken up by several cancer lines and demonstrated PDT-induced toxicity, with subcellular localization in the endoplasmic reticulum [15].

Most *in vitro* screening assays for PDT use two-dimensional monolayer cell cultures, and after promising leads are identified, *in vivo* animal models can be used. Compared to two-dimensional cell culture models, which has contributed to our knowledge of tumor biology and treatment effects, cells grown in a three-dimensional spheroid model better resemble many of the features found in tumors *in vivo* [16]. Since monolayers represent a highly artificial cellular environment and lack the three-dimensional aspects of a tumor [17–20], they are less reliable in predicting effectiveness of treatments *in vivo*. Moreover, human solid tumors are heterogeneously oxygenated and contain hypoxic regions that can lead to necrosis [21]. Hypoxia and necrosis in progressive solid tumors generate heterogeneities that result in populations of cancer cells more resistant to ROS-dependent radiation [22], and chemotherapy [19]. Because PDT relies on an oxygen-dependent cytotoxicity, a monolayer model is not sufficient to predict or understand what happens *in vivo*. To address this limitation, researchers have turned to a more complex model using spheroids [1, 23, 24]. Spheroids can closely resemble the properties of tumors because they are three-dimensional cell aggregates with a necrotic core surrounded by a shell of viable cells. This is particularly important because it allows for the formation of a gradient of nutrients, oxygen, and differential gene expression that can influence PDT efficacy. Spheroids of cancer cells were used to study the effectiveness of zinc sulfophthalocyanine [8, 25], aluminum-phthalocyanine chloride [9] and methylene blue [1]; however, these studies neither used dyes appended with cancer targeting motifs such as sugars, nor probed specific metabolic alterations occurring during spheroid formation. PDT efficacy with glycosylated PS (specifically those conjugated with glucose or galactose) is highly dependent on metabolism of cancer cells, the redox state of the cell, and expression of proteins such as galectin-1 and glucose transporter 1 (GLUT1). Thus, the present study develops an *in vitro* three-dimensional spheroid model using human cancer cell lines from different origins for evaluation of glycosylated PS. We demonstrate that spheroids display significant differences in glucose metabolism, endogenous ROS levels, galectin-1 and GLUT1 protein levels, so give a more accurate prediction of PDT efficacy compared with corresponding two-dimensional monolayers cultured on flat and rigid substrates.

## Materials and methods

### Glycosylated porphyrin

PorGlu_4_, 5,10,15,20-tetrakis-(4–1’-thio-glucosyl-2,3,5,6-tetrafluorophenyl)porphyrin was synthesized as previously described [13] and a stock solution of PorGlu_4_ was prepared at a concentration of 2 mM in dimethyl sulfoxide (DMSO; Sigma-Aldrich, St Louis, MO, USA). Fresh working solutions of PorGlu_4_ 2.25–9.0 μM were prepared in sterile phosphate-buffered saline (PBS) keeping the concentration of DMSO less than 0.5% (v/v).

### Monolayers cultures

HCT-116 colon cancer cells, MCF-7 and MDA-MB-231 breast cancer cells, UM-UC-3 bladder cancer cells, and HeLa cervical cancer cells were obtained from the American Type Culture Collection (ATCC^®^, Manassas, VA, USA). All batches of culture media were supplemented with 10% (v/v) of fetal bovine serum (Life Technologies, Carlsbad, CA, USA), 100 U/mL penicillin, 100 μg/mL streptomycin and 0.25 μg/mL amphotericin B (Sigma). HCT-116 colon cancer cells, MCF-7 and MDA-MB-231 breast cancer cells were cultured in Dulbecco´s Modified Eagle´s Medium (DMEM, Sigma). UM-UC-3 bladder cancer cells were cultured in Eagle´s Minimum Essential Medium (EMEM; Corning, NY, USA) with 1.5 g/L sodium bicarbonate, non-essential amino acids, L-glutamine and sodium pyruvate. HeLa cervical cancer cells were cultured in DMEM (Corning) with 4.5 g/L glucose, and L-glutamine without sodium pyruvate. All cells were maintained at 37°C in a 5% CO_2_ humidified atmosphere.

### Spheroid cultures

Spheroids were generated by growing cancer cell suspensions in agarose-coated 96 well plates [2, 18]. The agarose prevented the cells from attaching to the bottom of the wells. Briefly, 1.5% (w/v in PBS) agarose solution (MP Biomedicals, Santa Ana, CA, USA) was added to the wells of a 96-well microplate. Next, cells were seeded at densities ranging from 2,500 to 20,000 cells per well and the microplate was then gently spun by hand. Clusters of cancer cells were observed after 24 h of seeding. For HCT-116, MCF-7, UM-UC-3 and HeLa cancer cells, it took nearly 48 h for these clusters to form spheroids (*i*.*e*. clusters which are not dislodged by pipetting). MDA-MB-231 breast cancer cells did not form spheroids under these conditions for at least 72 h after cell seeding. The spheroids formed under these conditions are a specific characteristic of cancer cells, since a non-tumoral cell line, BJ-hTER primary fibroblasts, did not form viable spheroids for at least 72 h after cell seeding (data not shown).

Images of spheroids were obtained at 24, 48 and 72 h after cell seeding using the image analysis system consisting of Nikon Eclipse Ti fluorescent microscope and an Andor iXon EMCCD camera. Volume and surface area were calculated using Optimas^®^ image analysis software (Optimas, USA; version 5.0). The size of nearly 30 spheroids was calculated for each cell line [22] by measuring two orthogonal diameters (d1 and d2) using the line morphometry function. Volumes were calculated using the formula: volume = 4/3πr^3^, where r = 1/2√d1d2 is the geometric mean radius. Spheroids with volume ≈0.05 mm^3^ were obtained 48 h after plating 5,000 HCT-116 cells per well, 15,000 MCF-7 cells per well, 20,000 UM-UC-3 cells per well and 20,000 HeLa cells per well. These conditions were used to obtain spheroids for the determination of the doubling time, glucose utilization, lactate production, preparation of cells extracts, ROS determination, uptake and PDT assays. Average cell number per spheroid was determined at 24, 48 and 72 h after cell seeding by trypsinizing six different spheroids, mixing the cell suspension with Trypan blue (Sigma) and counting the number of viable cells. The total number of cells obtained was divided by the number of trypsinized spheroids.

The spheroid doubling times were determined by direct measurement of cell numbers, as described above. The spheroids were harvested at 24, 48 and 72 h after seeding cells in agarose-coated 96-well plate. Doubling times were determined using the formula: N/N_0_ = e^kt^ [26], where N is the cell number for a spheroid at a certain time (t) and N_0_ is the corresponding cell number at time zero. The constant k was calculated for each spheroid by plotting ln(N/N_0_) *versus* t, between 24 and 72 h the period of time in which the cell growth rate was linear. The doubling time was then determined using the above formula and N/N_0_ = 2.

### Estimation of glucose utilization and lactate production

Glucose utilization in monolayer and spheroid cultures was determined using the glucose uptake cell-based assay kit (Cayman Chemical, Ann Arbor, MI, USA). The spheroids were obtained 48 h after plating cells in agarose-coated black, clear bottom 96-well plate. Monolayer and spheroid cultures were incubated in PBS for 2 h. The cultures were then incubated with 100 μg/mL of fluorescently-tagged glucose derivative (2-deoxy-2-[(7-nitro-2,1,3-benzoxadiazol-4-yl)amino]-D-glucose, 2-NBDG) in PBS for 1 h. The supernatant was removed and cell cultures were washed with cell-based assay buffer. After removal of supernatant, 100 μL of cell-based assay buffer was added to each well and 2-NBDG taken up by cells was detected in a Gemini EM Microplate Spectrofluorometer (Molecular Devices, Sunnyvale, CA, USA) with the excitation and emission filters set at 485 nm and 535 nm, respectively.

Lactate production in monolayer and spheroid cultures was determined using the colorimetric lactate assay kit (Sigma). The spheroids were obtained 48 h after plating cells in agarose-coated 96-well plate. After incubation of monolayer and spheroid cultures with PBS for 2 h, 50 μL of supernatant was collected to a new plate and mixed with 50 μL of master reaction mix (46 μL lactate assay buffer, 2 μL lactate enzyme mix, 2 μL lactate probe) during 30 min at room temperature. The lactate produced was detected by measuring the absorbance at 570 nm in a PowerWave HT Microplate Spectrophotometer (ThermoFisher Scientific, Waltham, MA, USA).Cells were lysed in 1% (m/v) sodium dodecyl sulfate (SDS, Sigma) solution in PBS (pH 7.0) and the protein concentration was determined by bicinchoninic acid reagent (Pierce, Rockford, IL, USA). Glucose consumed or lactate produced was normalized to protein concentration.

### Detection of intracellular reactive oxygen species (ROS) generation

Monolayer and spheroid cultures growing in black, clear bottom 96-well plate were washed twice with PBS and incubated with 5 μM of 2′,7′-dichlorodihydrofluorescein diacetate (H_2_DCFDA; Invitrogen Life Technologies, Carlsbad, CA, USA) for 1 h, at 37°C, protected from light. After incubation, cells were washed with PBS and DCF (oxidized H_2_DCFDA) fluorescence was determined using a microtiter plate reader (Gemini EM Microplate Spectrofluorometer) with the excitation and emission filters set at 485±20 nm and 528±20 nm, respectively. Cells were then lysed in 1% (m/v) SDS solution in PBS (pH 7.0) and protein concentration was determined using the Pierce^®^ BCA Protein Assay Kit. ROS production was normalized to protein concentration.

### Preparation of cell extracts and western blot

Scraped monolayer cells and spheroids cultured for 48 h were collected at 1,500 g for 5 min, washed twice with ice-cold PBS and whole protein lysates were extracted using RIPA buffer (150 mM NaCl, 50 mM Tris-HCl, pH 7.5, 5 mM ethyleneglycol tetra-acetic acid, 1% Triton X-100, 0.5% sodium deoxycholate, 0.1% SDS, 2 mM phenylmethanesulfonyl, 2 mM iodoacetamide, and 1x protease inhibitor cocktail (Roche, Indianapolis, IN, USA)). Cell extracts were centrifuged at 16,000 g for 10 min at 4°C. Supernatants were used for protein quantification using the Pierce^®^ BCA Protein Assay Kit, followed by denaturation of the sample with Laemmli buffer. For the Western Blot analysis, 60 μg proteins were loaded per lane on sodium dodecyl sulphate-polyacrylamide gels (SDS-PAGE). Following electrophoresis and transfer to polyvinylidene fluoride membranes (Bio-Rad, Hercules, CA, USA), the blots were incubated in 5% nonfat milk in TBS-T (20 mM Tris, 150 mM NaCl, Tween 0.2%, pH 7.6). Membranes were then incubated with rabbit anti-Galectin1 1:5,000 (Thermo Fisher Scientific), rabbit anti-GLUT1 1:1,000 (Thermo Fisher Scientific) and rabbit anti β-actin 1:1,000 (Thermo Fisher Scientific) antibodies. After washing, the membranes were incubated with IRDye^®^ 800CW anti-Rabbit IgG 1:15,000 (LI-COR Biosciences, Lincoln, NE, USA) and imaged on the Odyssey Infrared Imaging System (LI-COR Biosciences) followed by densitometric analysis.

### Cellular uptake of PorGlu_4_

After incubation of monolayer or spheroid cultures with PorGlu_4_ in the dark, cells were immediately washed with PBS buffer and mechanically scraped in 1% (m/v) SDS in PBS buffer at pH 7.0. Spectrofluorometric determination of the intracellular concentration of PorGlu_4_ used a microplate reader (Gemini EM Microplate Spectrofluorometer) with the excitation and emission filters set at 410 nm and 702 nm, respectively, and the results were normalized for protein concentration using the Pierce^®^ BCA Protein Assay Kit.

For microscopic evaluation, cells were grown on coated glass coverslips with poly-L-lysine (Sigma). The cells were incubated with 9 μM PorGlu_4_ for 4 h, at 37°C. After incubation, cells were washed twice with PBS and fixed with 4% paraformaldehyde (PFA; Merck, Darmstadt, Germany) for 10 min at room temperature. The samples were then rinsed in PBS, and mounted in VectaSHIELD mounting medium containing 4´,6-diamidino-2-phenylindole (DAPI; Vector Laboratories, CA, Burlingame) for visualization under a confocal microscope (Nikon Eclipse Ti With Ultra High Speed Wavelength Source, Molecular Devices) equipped with Andor iXon EMCCD camera. For DAPI detection, specimen was excited at 405 nm and light emitted was collected with a 460/50 nm band pass filter. PorGlu_4_ fluorescence was obtained using 640 nm excitation and a 685/40 nm emission band pass filter.

### Photodynamic therapy (PDT) assay

Photodynamic irradiation was carried out after incubation of monolayer or spheroid cultures with 9 μM of PorGlu_4_ for 4 h. Cells were covered with fresh culture medium and exposed to light (420–700 nm) emitted by OLED Lumiblade Brite FL300 wm Level 4 (OLEDWorks, Rochester, NY, USA) delivered at 0.44 mW/cm^2^during 30 min (0.792 J/cm^2^). Sham-irradiated cells, used as controls, consisted in cells kept in the dark for the same durations and under the same environmental conditions as the irradiated cells. In all treatments, triplicate wells were established under each experimental condition, and each experiment was repeated at least three times. Cytotoxicity was determined in monolayer and spheroid cultures 24 h after treatment using the MTT and lactate dehydrogenase (LDH) assay, respectively.

### MTT and lactate dehydrogenase assays

Cell metabolic activity of cells growing in monolayers was determined by measuring the ability of cells to reduce 3-[4,5-dimethylthiazol-2-yl]-2,5-diphenyl-tetrazolium bromide (MTT, Sigma), to a colored formazan using a microplate reader (PowerWave HT Microplate Spectrophotometer). The data were expressed in percentage of control (*i*.*e*. optical density of formazan from control cells).

Unlike other studies reporting the use of MTT to study spheroid viability, this assay could not be applied to the evaluation of spheroids viability since the results were neither reproducible nor consistent (data not shown). Therefore, the CytoTox 96^®^ Non-Radioactive Cytotoxicity Assay (Promega, Madison, WI, USA) which measures lactate LDH was used to determine cytotoxicity in spheroid cultures. Briefly, 50 μL of culture medium was collected from the 96-well plate containing cell spheroids and mixed with 50μL of the CytoTox 96^®^ Reagent in dark for 30 min at room temperature. After incubation, 50 μL of stop solution was added to each well and the absorbance was recorded at 490 nm using a microplate reader (PowerWave HT Microplate Spectrophotometer). The average values of the culture medium background were subtracted from all values of experimental wells. The protein concentration was determined by Pierce^®^ BCA Protein Assay Kit after scrapping spheroid cultures in 1% (m/v) SDS solution in PBS (pH 7.0); and LDH activity was normalized to protein concentration. The results were normalized to the maximal LDH release, which was determined by treating the control wells for 60 min with 1% Triton X-100 to lyse all cells.

### Statistical analysis

Statistical analyses were carried out using a statistics program (GraphPad Prism; GraphPad Software). Student’s *t*-test was used to compare the treatment effects with that of control. P-value was considered at the 5% level of significance to deduce inference of the significance of the data. All graphs and statistics were prepared using the GraphPad Prism 5.0 software.

## Results

### Kinetics of spheroids growth

**Fig 1:**
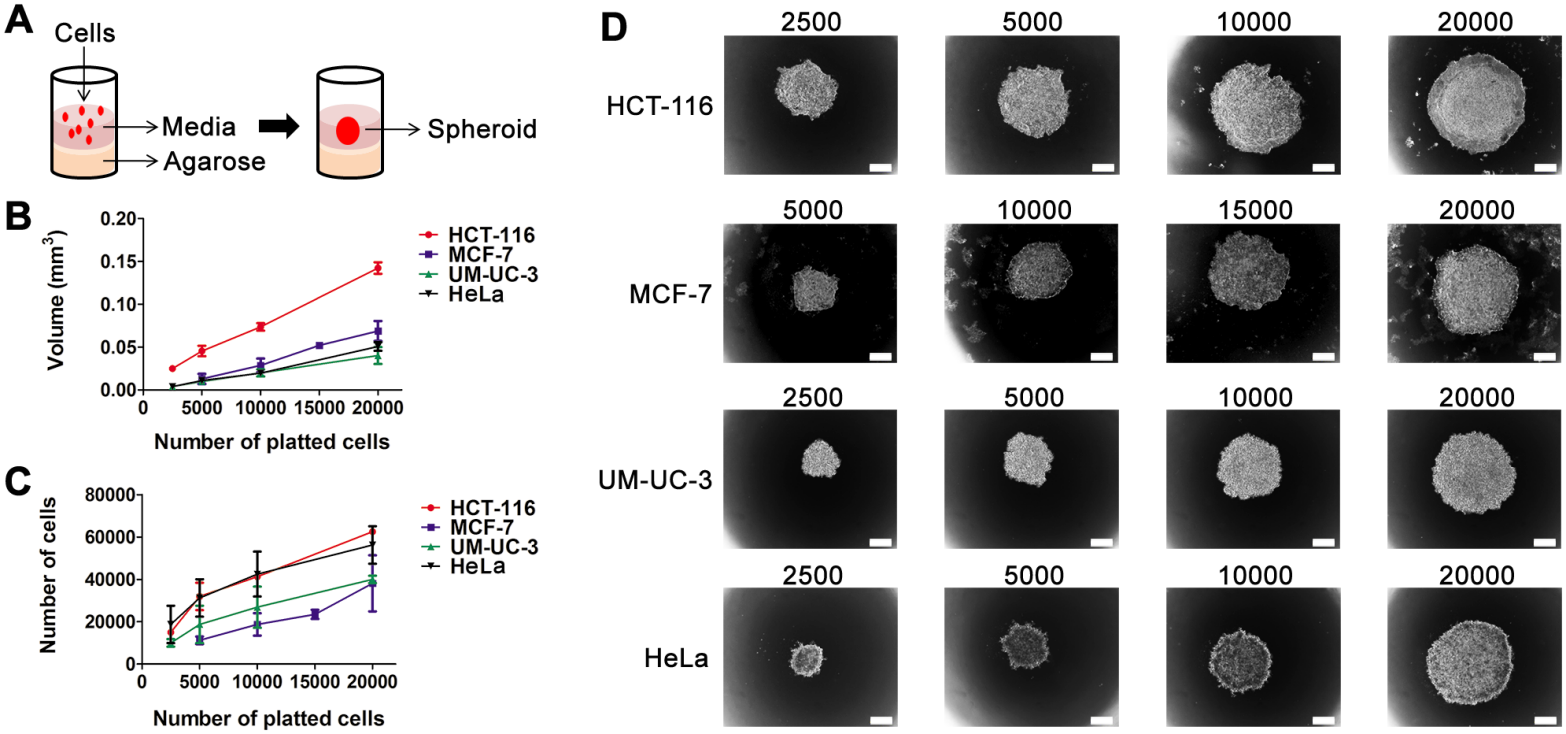
Spheroid size depends on cell seeding number. **A** spheroid plating scheme: one spheroid per well was formed in agarose-coated 96-well plates. **B, C** volume (mm^3^) and number of cells of HCT-116, MCF-7, UM-UC-3 and HeLa cancer cells at 48 h after plating with seeding densities of 2,500–20,000 cells per well for HCT-116, UM-UC-3 and HeLa cancer cells and 5,000–20,000 cells per well for MCF-7 cancer cells. Data are mean ± S.D. of at least 30 spheroids. **D** representative bright field images of spheroids at 48 h after plating with seeding densities of 2,500–20,000 cells per well for HCT-116, UM-UC-3 and HeLa cancer cells and 5,000–20,000 cells per well for MCF-7 cancer cells. *Scale bars* 100 μm.

The culture of certain types of cells on surfaces that are unfavourable for cell attachment led to enhancement of homotypic cell-cell interactions, resulting in formation of three-dimensional structures (spheroids, Fig 1A) [20]. In our study, five different cell types were tested for spheroid formation in agarose-coated wells of a 96-well plate (Table 1). The HCT-116 colon cancer cells, MCF-7 and MDA-MB-231 breast cancer cells, UM-UC-3 bladder cancer cells and HeLa cervical cancer cells were cultured in suspension on agarose bed which forms a thin non-adherent film and prevents cell attachment. Agarose is an efficient substrate to prevent cell attachment to the plate as reported in several studies describing spheroid cultures [2, 19, 27–29].

**Table 1:** Human cell lines tested for spheroids formation and their respective doubling times (h).

Cell line	Origin	Doubling time (h)	
		Monolayers (Reference)	Spheroids
HCT-116	Colon Cancer	17 [30]	19
MCF-7	Breast Cancer	25 [30]	38
UM-UC-3	Bladder Cancer	20 [31]	23
HeLa	Cervival Cancer	19 [30]	23
MDA-MB-231	Breast Cancer	42 [30]	N.D.

**Fig 2:**
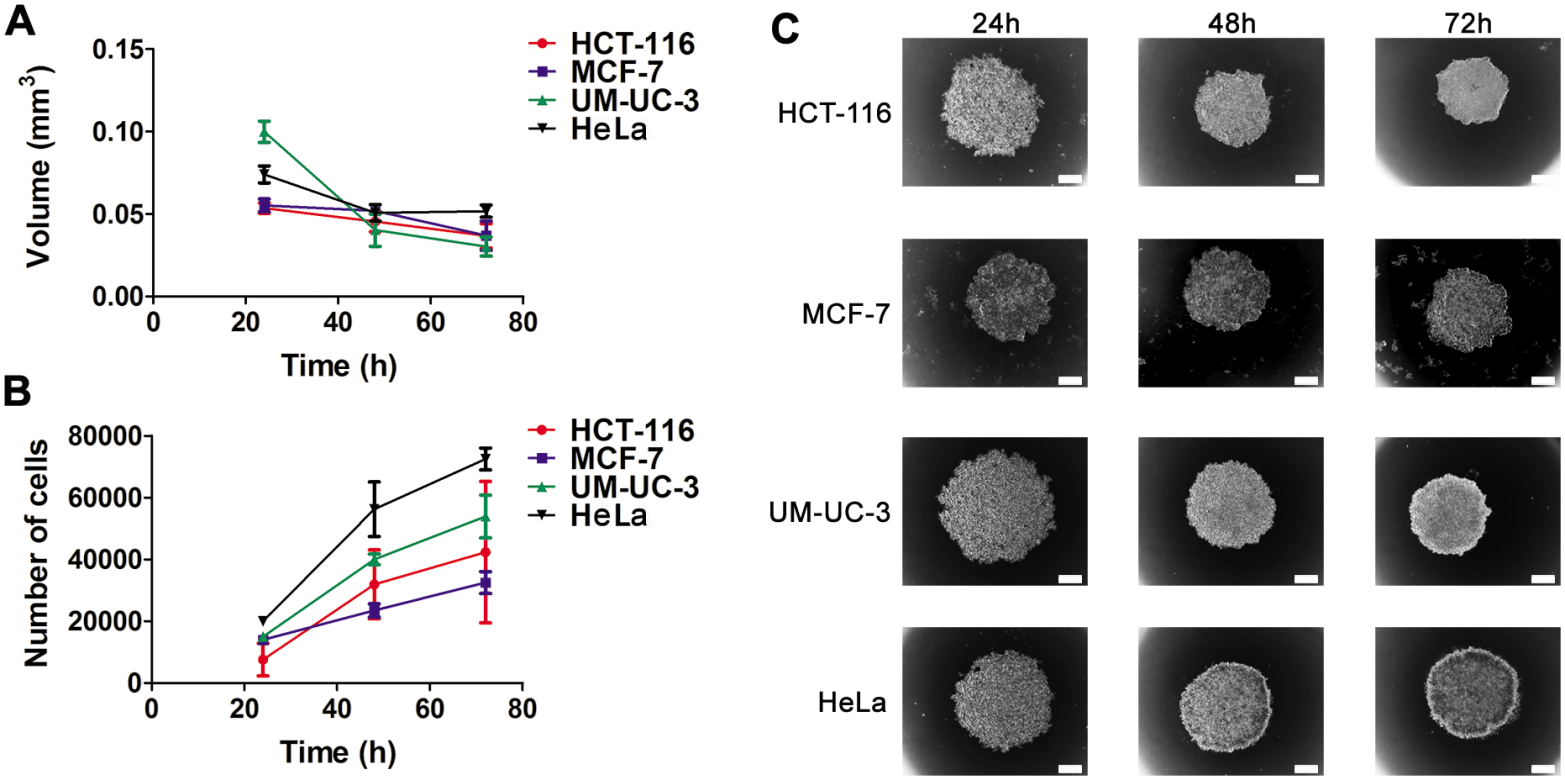
Spheroid size depends on time after cell seeding. **A, B** volume (mm^3^) and number of cells of HCT-116, MCF-7, UM-UC-3 and HeLa cancer cells at 24, 48 and 72 h after plating with seeding densities of 5,000 HCT-116 cells per well, 15,000 MCF-7 cells per well, 20,000 UM-UC-3 cells per well and 20,000 HeLa cells per well. Data are mean ± S.D. of at least 30 spheroids. **C**, representative bright field images of spheroids at 24, 48 and 72 h after plating with seeding densities of 5,000 HCT-116 cells per well, 15,000 MCF-7 cells per well, 20,000 UM-UC-3 cells per well and 20,000 HeLa cells per well. *Scale bars* 100 μm.

After seeding on the agarose-coated wells, cells started to cluster, and the first loose aggregates were already observed after 2 h of incubation. During the first 24 h of incubation, the cells formed aggregates which were easily dissociated by mechanical force. Most of the tumor cells tested (HCT-116, MCF-7, UM-UC-3 and HeLa) gave rise to compact, rigid and spherically-shaped spheroids (Figs 1 and 2) by 48 h after cell plating. The increase in the compactness of cancer cells from 24 h to 48 h after cell plating was accompanied by a decrease in the volume. When spheroid cultures were observed under phase contrast microscope, a multilayer cell assembly was observed. The absence of necrotic core formation in the spheroids was evident at all time points examined, indicative of cell viability in the interior of the spherical structures. These cultures were resistant to gentle agitation or physical transfer and only enzymatic digestion could separate these three-dimensional structures into single cells. MDA-MB-231 breast cancer cells remained as single suspended cells forming aggregates which were not stable enough for harvest and further experiments.

We analysed the kinetics of spheroid growth and the proliferation rate of HCT-116, MCF-7, UM-UC-3 and HeLa cancer cells (Figs 1 and 2, S1–S4 Tables). Fig 1D shows representative optical images of spheroids with different initial cell seeding density (2,500–20,000 cells per well). A higher cell density in the initial suspension gave rise to spheroids with larger volumes and number of cells per spheroid than lower cell densities (Fig 1B–1D). HCT-116, MCF-7, UM-UC-3 and HeLa spheroids showed similar growth pattern, *i*.*e*. the volume decreased significantly until 48 h in culture, then maintaining until 72 h with small fluctuations (Fig 2A and 2C). Between 48 to 72 h after cell plating, the border of the spheroids became ruffled, suggesting cell proliferation at the spheroid periphery (Fig 2C). The number of viable cells per spheroid increased with time after cell seeding (Fig 2B) with greater doubling times compared with monolayer cultures (Table 1).

In an attempt to use spheroids with similar volume in our studies to guarantee the homogeneity of our three-dimensional populations, we determined the cell seeding densities by which spheroids of different cell lines can be obtained with similar size after 48 h in culture (S1–S4 Tables, Fig 2A). The culture of 5,000 HCT-116 cells per well, 15,000 MCF-7 cells per well, 20,000 UM-UC-3 cells per well, 20,000 HeLa cells per well gave rise to spheroids with volume and average size of approximately 0.05 mm^3^and 443 μm, respectively.

### Spheroid cells are viable with altered patterns of metabolism

**Fig 3:**
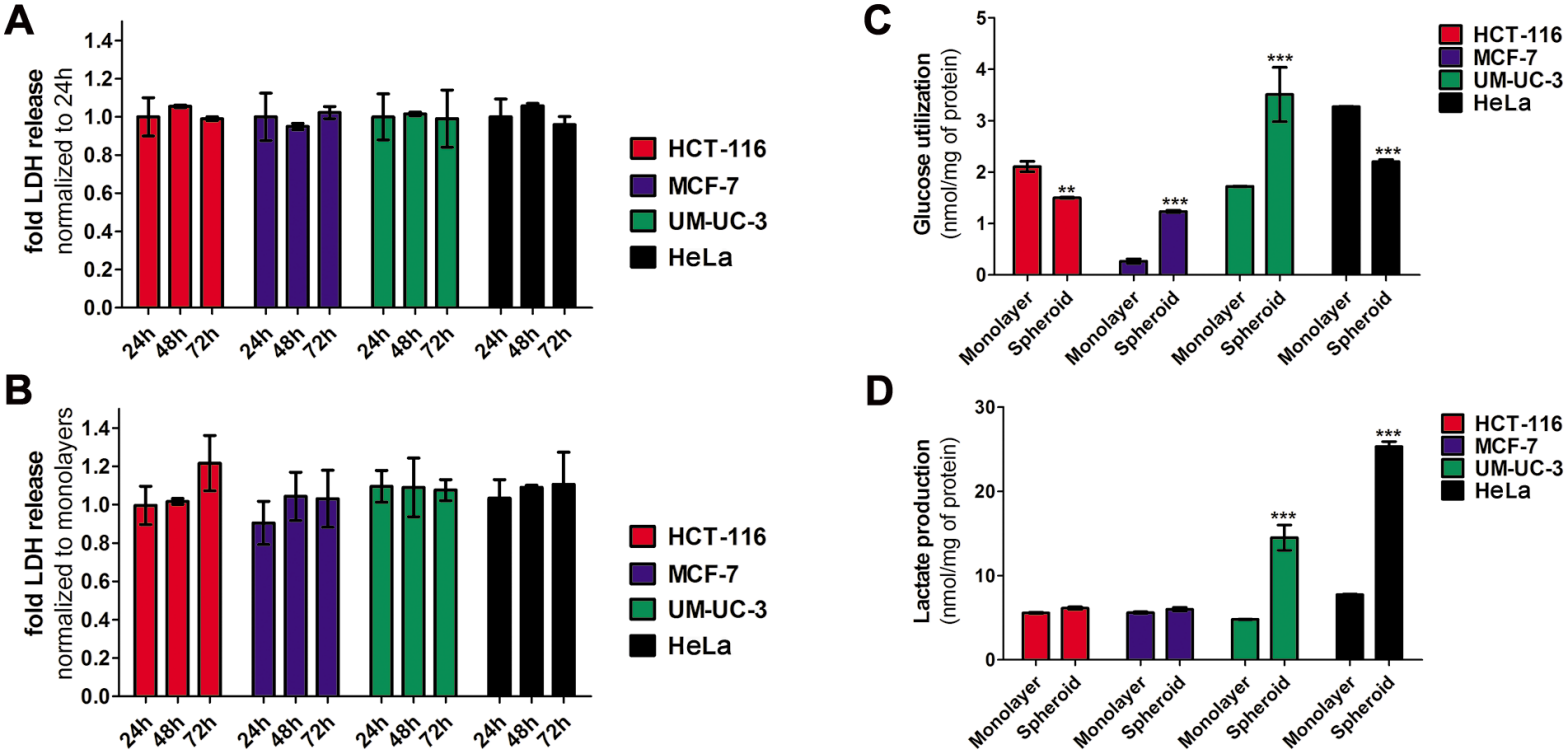
Spheroid cells are viable and have altered metabolism. **A, B** spheroids viability at 24, 48 and 72 h after plating with seeding densities of 5,000 HCT-116 cells per well, 15,000 MCF-7 cells per well, 20,000 UM-UC-3 cells per well and 20,000 HeLa cells per well. Viability was determined using the LDH assay. LDH activity was measured in the culture supernatant and data were normalized to LDH activity of spheroids at 24 h after cell plating (**A**) or LDH activity of cells growing in monolayers (**B**). Data are means ± S.D. of at least three independent experiments performed in triplicate. **C, D** glucose utilization and lactate production of cancer cells growing in monolayers and in spheroids at 48h after plating with seeding densities of 5,000 HCT-116 cells per well, 15,000 MCF-7 cells per well, 20,000 UM-UC-3 cells per well and 20,000 HeLa cells per well. The amount of glucose (nmol, **C**) and lactate (nmol, **D**) were estimated using enzymatic assays and the results were normalized for mg of protein. Data are means ± S.D. of at least three independent experiments performed in triplicate. **P< 0.01, ***P< 0.001 compared to glucose utilization or lactate production of the respective cancer cell line growing in monolayers.

To assess cellular viability of spheroid cultures, the MTT cell metabolic activity assay was performed on intact or dissociated spheroids [21, 32], but this assay did not result in reproducible and consistent results (data not shown). Determination of cell death *via* measurement of molecules released from membrane-defective cells into the extracellular medium demonstrated to be preferable to evaluate spheroids viability. Samples of the media from monolayer or spheroid cultures were used to estimate the amount of lactate LDH release by cells that have damaged membranes due to necrosis or apoptosis [32]. The amounts of LDH released by spheroids at 48 and 72 h were similar to those at 24 h after cell plating (Fig 3A) and to monolayer cultures (Fig 3B), thereby demonstrating that the spheroids are viable structures for at least 72 h after cell plating.

Spheroids are high cell-density, closely packed three-dimensional structures that more closely simulate conditions existing in under-perfused solid tumors where hypoxia and related alterations in cellular metabolism occur due to increasing distances from the nourishing blood capillaries. Since the diffusion of molecules such as glucose and oxygen can be limited, there are lower concentrations in the inner regions of spheroids [22, 33]. The glycolytic flux of cells can be determined by measuring glucose consumption and lactate production [34]. To determine the glucose consumption, we incubated the cells with the fluorescent glucose analog 2-NBDG for 1 h and the intracellular accumulation was measured by fluorescence spectroscopy. The glucose utilization (nmol per mg of protein) in monolayer cultures decreased in the following order HeLa > HCT-116 > UM-UC-3 > MCF-7 (S1 Fig). The glucose consumption by MCF-7 and UM-UC-3 spheroids was greater than the corresponding cells growing in monolayers (Fig 3C). Conversely, spheroids of HCT-116 and HeLa demonstrated lower glucose consumption than corresponding monolayer cultures. Lactate production (nmol/mg of protein) was determined in the culture medium using a commercially available colorimetric kit. The lactate production in monolayer cultures decreased in the following order: HeLa > HCT-116 ≈ MCF-7 >UM-UC-3 (S2 Fig). A significant increase in lactate production was observed from UM-UC-3 and HeLa spheroids (Fig 3D), but no alterations in lactate production were observed during HCT-116 and MCF-7 spheroid development.

### Protein levels of galectin-1 and GLUT1 proteins are altered in spheroids

**Fig 4:**
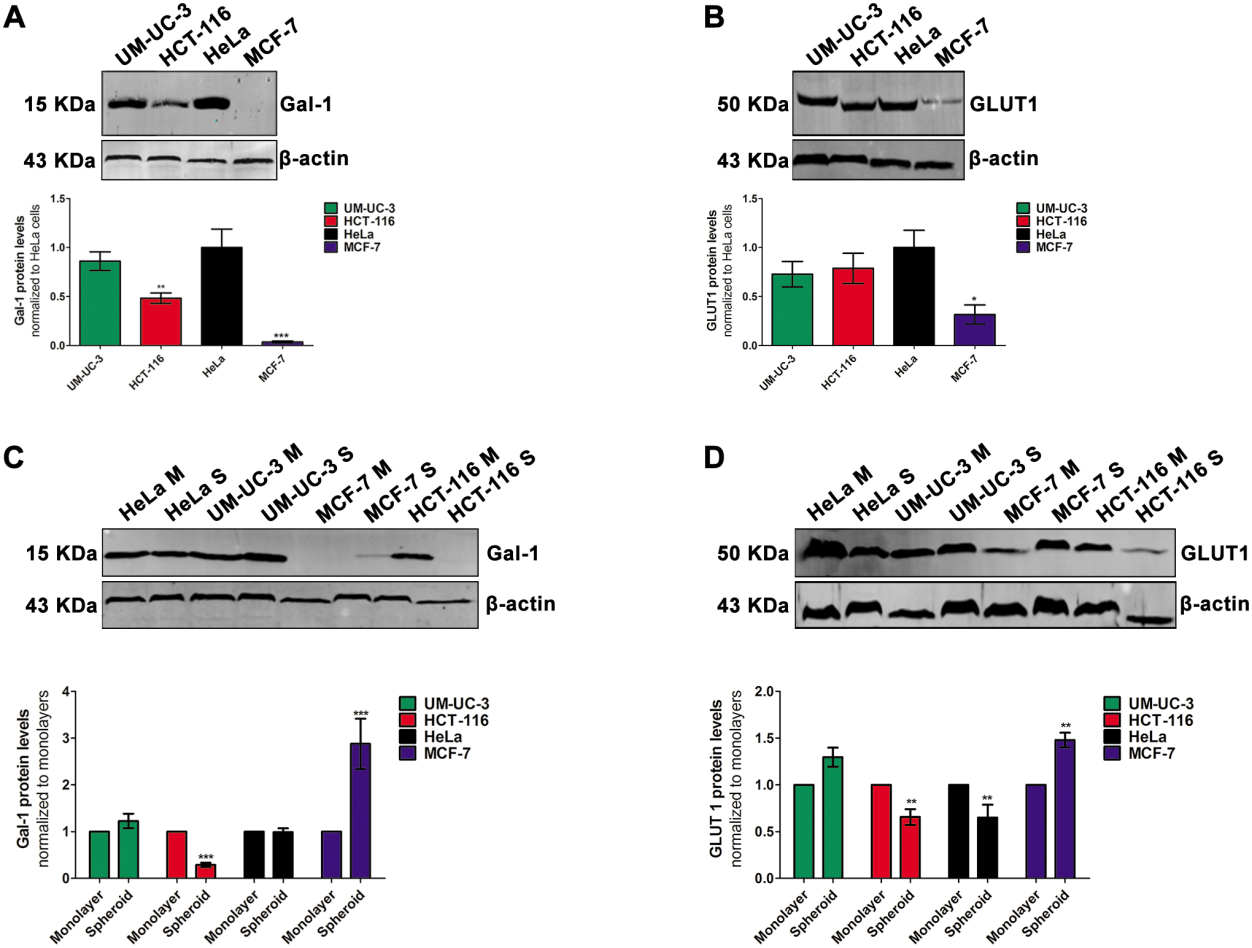
Spheroid cells have altered galectin-1 and GLUT1 protein levels. **A, C** western blot analysis and quantification of galectin-1 protein levels in monolayers of HCT-116, MCF-7, UM-UC-3 and HeLa cancer cells growing in monolayers (**A**) or in spheroids (**C**). M means monolayer and S, spheroid. Quantitative analysis of galectin-1 (normalized to β-actin) expressed as a ratio of the levels found in HeLa cells (**A**) or in cancer monolayers (**C**). Data are means ± S.D. of at least five independent experiments. ***P< 0.001 compared to galectin-1 protein levels in HeLa cells (**A**) or in respective cancer cell line growing in monolayers (**C**). **B, D** western blot analysis and quantification of GLUT1 protein levels in monolayers of HCT-116, MCF-7, UM-UC-3 and HeLa cancer cells growing in monolayers (**B**) or in spheroids (**D**). M = monolayer and S = spheroid. Quantitative analysis of GLUT1 (normalized to β-actin) expressed as a ratio of the levels found in HeLa cells (**B**) or in cancer monolayers (**D**). Data are means ± S.D. of at least five independent experiments. *P< 0.05, **P< 0.01 compared to GLUT1 protein levels in HeLa cells (**B**) or in respective cancer cell line growing in monolayers (**D**).

There is growing interest in the development of porphyrinoids conjugated with sugars such as glucose and galactose as new PSs for the PDT of cancer [6, 10, 11, 13, 35, 36]. Galectin-1 and GLUT1 are glyco-binding proteins overexpressed in cancer cells [37, 38] that play a key role in the uptake and photo-toxicity of with galactose- and glucose- porphyrin conjugates [11, 39, 40]. We evaluated the expression levels of these proteins in monolayers of HCT-116, MCF-7, UM-UC-3 and HeLa cancer cells, by Western blots (Fig 4A and 4D). To determine whether the expression of these proteins is altered during spheroids development, we compared these results to cellular extracts of spheroid cultures (Fig 4C and 4D). Both galectin-1 and GLUT1 expression was higher in MCF-7 spheroids compared to monolayer cultures. Whereas no alterations in galectin-1 expression was observed during growth of HeLa and UM-UC-3 spheroids, the expression was lower in spheroids of HCT-116. GLUT1 protein levels were lower in spheroids of HCT-116 and HeLa. The galectin-1 protein levels in spheroids decreased in the following order: UM-UC-3 > HeLa > MCF-7 > HCT116 (S3 Fig). GLUT1 protein levels in spheroids decreased in the following order: MCF-7 > UM-UC-3 > HeLa ≈ HCT-116 (S4 Fig).

### PorGlu_4_ accumulates in both monolayer and spheroid cultures and is non-toxic

**Fig 5:**
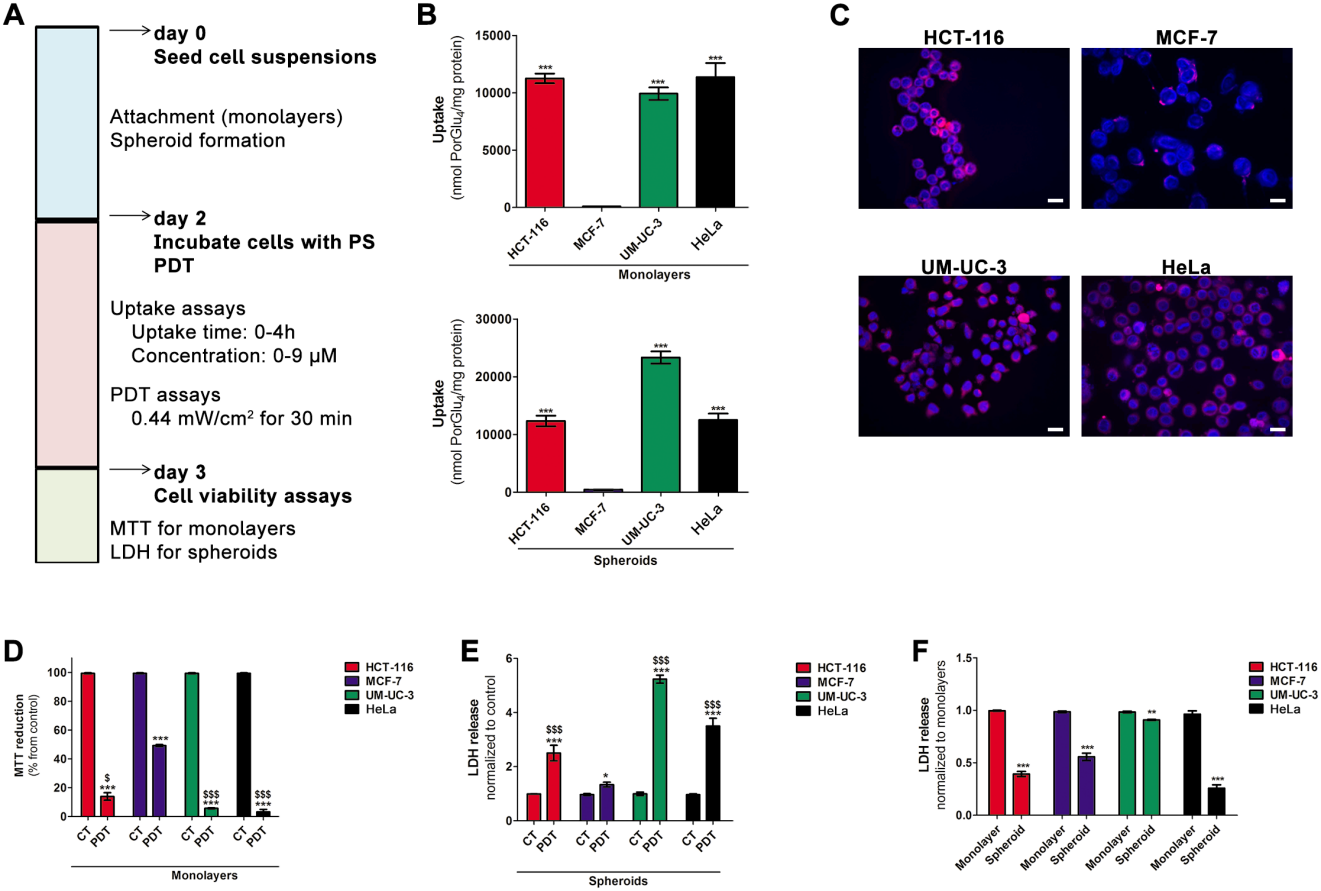
PorGlu_4_ produces toxicity after PDT in cancer cells growing as monolayers or spheroids. **A** uptake and PDT studies schedule. **B** intracellular uptake of PorGlu_4_ by HCT-116, MCF-7, UM-UC-3 and HeLa cancer cells growing as monolayers or spheroids. The concentration of PorGlu_4_ was determined by fluorescence spectroscopy (λ_excitation_ at 410 nm and λ_emission_ at 702 nm) after incubation of cancer cells with 9 μM of PorGlu_4_ for 4 h and the results were normalized to protein quantity. Data are means ± S.D. of at least three independent experiments performed in triplicate. ***P< 0.001 compared to PorGlu_4_ uptake by MCF-7 cancer cells. **C** representative fluorescence images of cancer cells incubated in darkness with 9 μM of PorGlu_4_ for 4 h (red, λ_excitation_ at 640 nm and λ_emission_ 685/40 nm band pass) and cell nucleus stained with DAPI (blue, λ_excitation_ at 405 nm and λ_emission_ at 460/50 nm band pass). *Scale bars* 20 μm. **D** cytotoxicity at 24 h after PDT with PorGlu_4_ in cancer cells growing in monolayers, determined using the MTT assay. Cells were incubated with 9 μM PorGlu_4_ for 4 h and PDT was performed during 30 min at 0.44 mW/cm^2^. Data are means ± S.D. of at least three independent experiments performed in triplicate. ***P< 0.001 compared to phototoxicity in control cells. ^$^*P*< 0.05, ^$ $ $^*P*< 0.001 compared to photoxicity in MCF-7 cancer cells. **E** cytotoxicity at 24 h after PDT with PorGlu_4_ in cancer cells growing in spheroids, determined using the LDH assay. Spheroids were incubated with 9 μM PorGlu_4_ for 4h and PDT was performed during 30 min at 0.44 mW/cm^2^. Data are means ± S.D. of at least three independent experiments performed in triplicate. *P< 0.05, ***P< 0.001 compared to phototoxicity in control spheroids. ^$ $ $^*P*< 0.001 compared to photoxicity in MCF-7 cells. **F** cytotoxicity at 24 h after PDT with PorGlu_4_ in cancer cells growing in monolayers and spheroids, determined using the LDH assay. Spheroids were incubated with 9 μM PorGlu_4_ for 4h and PDT was performed during 30 min at 0.44 mW/cm^2^. Data are means ± S.D. of at least three independent experiments performed in triplicate. **P< 0.01, ***P< 0.001 compared to phototoxicity in the respective monolayer culture.

Resistance of cancer cells to PDT agents arises from decreased PS photoactivity *in vivo*, PS delivery barriers, and the kinetics of tumor penetration by the PS. Since the three-dimensional structure of spheroids mimics some of the physical and physiological barriers found *in vivo* [19, 20, 24], spheroids of HCT-116, MCF-7, UM-UC-3 and HeLa were assessed as more predictive screens for the PDT activity of glyco-PSs. We performed uptake and PDT studies (Fig 5A) with a previously reported porphyrin conjugated with four glucose sugars (S5 Fig) [13].

Monolayer cultures of cancer cells were incubated 48 h after plating, with increasing concentrations (2.25, 4.5 and 9 μM) of PorGlu_4_ in PBS (containing a maximum of 0.5% v/v DMSO) for up to 4 h. No toxicity was observed in untreated cells for up to 4 h in the presence of 0.5% (v/v) DMSO in the incubation medium (S6 Fig). Moreover, PorGlu_4_ showed no significant dark cytotoxicity in monolayer and spheroid cultures at concentrations up to 9 μM (S7 Fig). We evaluated PorGlu_4_ accumulation in monolayer and spheroid cultures using fluorescence spectroscopy (Fig 5B, S8 and S9 Figs). The rate of uptake of 9 μM PorGlu_4_ for 4 h by monolayers of cancer cells was also evaluated by confocal fluorescence microscopy (Fig 5C). Initially, PorGlu_4_ is taken up quite readily by cancer cells, followed by a slower rate after 2 h (S8 Fig). The uptake of PorGlu_4_ by cancer cells depends on the concentration, and in monolayer cultures was observed to decrease in the following order: HeLa ≈ HCT-116 > UM-UC-3 > MCF-7 (Fig 5B and 5C). We compared the uptake of PorGlu_4_ into monolayer cultures *versus* spheroids (Fig 5B and S8 Fig) with a non-toxic concentration of PorGlu_4_ (S10 Fig). Spheroid structures of HCT-116 or HeLa cells had the same uptake of PorGlu_4_, but the uptake was *greater* for spheroids of MCF-7 and UM-UC-3 cancer cells than for monolayers.

### Photodynamic activation of PorGlu_4_ induces cytotoxicity in monolayers and spheroids

We investigated the effects of PorGlu_4_ mediated PDT efficacy in monolayer and spheroid cultures. For these studies we used human cancer cell line spheroids with volume and average diameter of 0.05 mm^3^ and 443 μm, respectively. The cells were treated 48 h after plating, with non-toxic PorGlu_4_ concentrations ranging from 2.25 to 9 μM for 4 h, and then exposed to light (420–700 nm) delivered at 0.44 mW/cm^2^ during 30 min (0.792 J/cm^2^). The 48 h time point following cell seeding was chosen based on the time needed for spheroids of the different cell lines to form and achieve reproducible and similar compactness (*vide supra*). In all monolayer cultures, the light activation of PorGlu_4_ induced a clear concentration-dependent reduction of cell viability (S11 Fig). As shown in Fig 5D, the phototoxicity induced by PorGlu_4_ in cell monolayers decreased in the following order: HeLa> UM-UC-3 > HCT-116 >MCF-7. When PDT was performed in spheroid cultures, the release of LDH into the cell culture medium was increased compared with controls (Fig 5E and S12 Fig). However, the amount of LDH leakage into the extracellular medium was lower for all cancer spheroid cultures compared to corresponding monolayers (Fig 5F).

ROS are primary mediators of PDT-induced cell death [5]. Previous studies reported alterations in endogenous ROS during spheroid development [22]. To determine endogenous as well as PDT-induced ROS generation in monolayers and spheroids, these cultures were stained with the redox sensitive dye H_2_DCFDA (2′,7′-dichlorodihydrofluorescein diacetate) which reacts with ROS and is oxidized into fluorescent DCF and monitored by fluorescence spectroscopy. Endogenous levels of ROS were similar in monolayers and spheroids of MCF-7 and UM-UC-3 cancer cells (S13 Fig), but for HCT-116 and HeLa spheroids there was greater endogenous ROS. PDT with PorGlu_4_ leads to ROS generation, which was greater in all monolayer cell lines compared to their respective spheroids (S14 and S15 Figs), even in the cases with a lower PS uptake.

## Discussion

The development of reliable *in vitro* models for the screening of anticancer agents also plays a key role in understanding cancer biology. Tumor spheroid cultures with appropriate size and shape simulate the three-dimensional tumor micro-milieu and *in vivo* environment in which cancer cells reside [19, 20], *e*.*g*. the physical-chemical conditions of oxygen, pH, and nutrient gradients. Some advantages of using spheroid cultures in PS screening are reported [1, 3, 4, 7–9, 17]; however, a high throughput screening that involves the use of different cancer cell lines is hampered by the difficulty of obtaining homogeneous spheroid cultures. The need to assay biochemical activity and viability of the spheroids for each culture is underappreciated. To analyze uptake and cytotoxicity of anticancer compounds using these three-dimensional tumor structures, it is necessary to obtain spheroids with a compact structure and reproducible diameters. Of the five tumor cells evaluated herein, HCT-116 colon cancer cells, MCF-7 breast cancer cells, UM-UC-3 bladder cancer cells and HeLa cervical cancer cells grew as viable, stable, structurally and functionally mature spheroids by 48 h after cell seeding. MDA-MB-231 breast cancer cells failed to form spheroids in agarose-coated 96-well plates, instead, formed loose aggregates.

The HCT-116, MCF-7, UM-UC-3 and HeLa spheroids formed 48 h after cell plating showed appropriate size for screening of PDT agents. Spheroids with a size smaller than 200 μm may be sufficient to mimic three-dimensional cell-cell and cell-matrix interactions but they are inappropriate to reflect pathophysiological conditions with hypoxic areas in the spheroid center or to mimic proliferation gradients [18]. Also, spheroids with diameter > 200 μm mimic the chemical gradients found *in vivo* [24]. Cells cultured in spheroids with sizes beyond 500 μm may become quiescent with formation of a necrotic core and undergo cell death due to limited diffusion of oxygen and nutrients inside spheroids [41, 42]. Studies have demonstrated that not only spheroid volume, but also spheroid shape may be a source of variability when these three-dimensional structures are used on the evaluation of a new anticancer compound [24]. Irregularly shaped spheroids are generally observed to have two necrotic cores instead of one, which results in inconsistent viability. Thus, we optimized conditions to obtain spherically-shaped spheroids of homogeneous size and preserved cell viability for at least 72 h after cell seeding.

Glycolysis converts glucose into pyruvate and then to lactic acid. In the presence of oxygen, most of the mitochondria of mammalian cells oxidize pyruvate to carbon dioxide and water, thereby inhibiting glycolysis [33]. The increased glycolysis in cancer cells leads to increased lactic acid, *i*.*e*. the Warburg effect, even if oxygen is available [43, 44]. High glucose consumption is a unique property of cancer cells and might have a key role in promoting tumor development [33]. Increased glucose transport in cancer cells is associated with increased expression of GLUTs (such as GLUT1 and GLUT3 [37, 45]) that contribute to the neoplastic process. Compared with tumors with typical oxygen levels, hypoxic tumors require increased glycolysis to survive [33], so the development of hypoxic regions in three-dimensional cultures increases glucose utilization and lactate production [46].

Previous studies demonstrated that glucose utilization and lactate production are higher in cancer cells spheroids compared with monolayers [22, 47]. Others reported that the glycolytic fluxes and GLUT1 proteins levels are dependent on spheroids age [48]. We observed alterations in glucose metabolism upon spheroid formation using the fluorescent glucose analog 2-NBDG which is taken into cells through glucose transporters [34, 49]. These results suggest that, concomitant to the development of MCF-7 and UM-UC-3 spheroid structures, there is alteration in the pattern of metabolism with increases in glucose dependence. Since the uptake of glucose by cancer cells depends on the levels of glucose transporters such as GLUT1 [33]; increased GLUT1 protein levels explain the increased glucose consumption by MCF-7 and UM-UC-3 spheroid cancer cells, and the uptake of PorGlu_4_. Interestingly, HCT-116 and HeLa spheroids demonstrated a decreased consumption of glucose arising from a decreased content of GLUT1. Previous studies demonstrated that dephosphorylation of 2-NBDG by hexokinase can result in efflux from cells [49]. Alterations in hexokinase expression have been observed during spheroids development [46], which for HCT-116 and HeLa spheroids can result in dephosphorylation and efflux of 2-NBDG and lead to an apparent decreased glucose consumption.

The commercial assay [34] reveals that the development of UM-UC-3 and HeLa spheroids results in increased lactate production relative to cell cultures, while lactate levels during the development of HCT-116 and MCF-7 spheroids were the same as the cell cultures. The increased lactate by UM-UC-3 spheroids likely arises from increased glycolysis. Previous studies demonstrated that lactate production and utilization can occur simultaneously and these processes are highly dependent on external lactate concentrations [50]. Therefore, the increased lactate in the culture medium of HeLa spheroids, in spite of decreased glucose utilization, can be due to alterations in lactate efflux [51]. The breakdown of cellular macromolecules or the catabolism of other fuels besides glucose can also give rise to the increased lactate levels found in the culture medium of HeLa spheroids [46]. Since lactate efflux depends on monocarboxylate transporter 1 (MCT1) [51], alterations in the expression of MCT1 during spheroids development can also explain the differences in lactate production. The differential lactate production in monolayers *versus* spheroids can also be a result of alterations in the expression of enzymes involved in lactate oxidation (such as LDH5 and LDH1 [52]).

The oxygen sensor transcription factor hypoxia-inducible factor-1α (HIF1α) has a key role in the glycolytic response [53]. Under hypoxic stress, the upregulation of HIF1α induces the expression of survival genes such as glucose transporters [54], haematopoeitic factors such as transferrin and erythropoietin [55], hexokinase II [56], and angiogenic growth factors such as vascular endothelial growth factor, VEGF. HIF1α expression is increased in spheroid cultures compared to cells growing in monolayers, additionally, HIF1α overexpression is accompanied by increased GLUT1 protein levels [48]. HIF1α overexpression increases galectin-1 expression and HIF1α inhibition attenuates hypoxia induction of galectin-1 [57]. Further, the enhanced glycolysis observed in UM-UC-3 and MCF-7 spheroids could be driven mainly by hypoxia, and induced by the increased expression of genes encoding proteins involved in glucose metabolism.

For screening of new glyco-PSs to be used in cancer PDT [6, 10, 58], the overarching hypothesis is that these dyes adhere to glyco-binding proteins such as galectin-1 and GLUT1 overexpressed in cancer cells, thereby improving the selectivity of the PS for cancer. In both MCF-7 and UM-UC-3 cancer cells, we found increased protein levels of galectin-1 and GLUT1 in spheroids compared to their respective monolayers. In HCT-116 spheroids the expression of these proteins was lower when compared with monolayer cultures. Galectin-1 protein content was unaltered in HeLa spheroids, while GLUT1 protein levels were reduced. The precise mechanism underlying these changes is protein expression in spheroids requires further investigation, yet it is well known that the expression of both galectin-1 and GLUT1 is altered during hypoxic conditions due to upregulation of HIF1α [54, 57]. Thus as an assay for the potential clinical value of glyco-PS as effective PDT agents, monolayer cultures of some cell lines can give over optimistic prognoses, while other cell lines underestimate the prospects.

Herein we used cell lines growing in monolayer and spheroid cultures, each expressing different levels of galectin-1 and GLUT1. The photophysical properties of the porphyrins allow us to correlate the expression of these proteins to PorGlu_4_ uptake and the phototoxicity in both monolayer and spheroid cultures. Because the tetraglycosylated porphyrin PorGlu_4_ and related glycosylated porphyrinoids are well-studied by several labs [13], it is an ideal system to validate the use of HCT-116, MCF-7, UM-UC-3 and HeLa spheroids in the *in vitro* studies of PDT with a glyco-PS.

The uptake and PDT phototoxicity with PorGlu_4_ depends on the cell line and on the *in vitro* model. Differences in uptake and localization of PorGlu_4_ in monolayers *versus* spheroids can result in different modes of cell death. Since cell death involves several complex processes of cellular damage, differential gene expression profiles in monolayers *versus* spheroids [16] can reveal different cell death pathways induced by PorGlu_4_ in these *in vitro* models. Since PorGlu_4_ corresponds to a porphyrin surrounded by four glucoses, it is expected that proteins able to bind glucose (such as GLUT1) have a key role during its uptake. As expected, the uptake of PorGlu_4_ was greater in cell lines expressing higher levels of GLUT1 proteins than in cell lines with lower levels of these proteins. Interestingly, galectin-1 is unlikely to be involved in the uptake of PorGlu_4_, since a decreased expression of this protein in HCT-116 compared with UM-UC-3 and HeLa cancer cells did not correspond to a proportional decreased uptake. Although the increased levels of GLUT1 proteins during the development of MCF-7 and UM-UC-3 spheroids resulted in increased PorGlu_4_ uptake, the three-dimensional culture model demonstrated greater resistance to phototoxicity compared with monolayer cultures, which is consistent with previous studies [1]. Many factors affect PS accumulation in spheroids, and hypoxia and related alterations in the metabolism affect the phototoxic response. Additionally, the slower proliferation of spheroids compared with monolayer cultures can contribute to the lower phototoxicity in spheroids. In monolayer cultures, the cells are exposed to uniform PorGlu_4_ and oxygen levels. For spheroids, the reduced diffusion of PorGlu_4_ and oxygen to the center of spheroids reduces the ROS production, thereby reducing PDT efficacy in the core. Although previous studies reported the formation of a necrotic core in spheroid development due to lack of nutrition and oxygen [20], we did not observe this phenomenon when imaged in bright-field microscopy [24] because we used 423–463 μm spheroids cultured for short times (72 h).

Changes in cell redox status with increasing endogenous generation of ROS typically are observed during spheroid development [22]. Alterations in spheroids redox status (*e*.*g*. activity of antioxidant enzymes) can be involved in the resistance of spheroid cultures compared with cells growing in monolayers. We detect an increase in endogenous ROS generation during the development of HCT-116 and HeLa spheroids. The GLUT1 protein has antioxidant functions in cells due to its ability to import the oxidized form of vitamin C (dehydroascorbic acid) [59] to the mitochondria. Therefore, it is expected that the decrease in GLUT1 protein expression in HCT-116 and HeLa spheroids results in an increase of endogenous ROS. We confirmed that the phototoxicity induced in monolayers and spheroids after PDT with PorGlu_4_ was mediated by the generation of ROS. As expected, the ability of PorGlu_4_ to induce ROS generation was greater in HCT-116, UM-UC-3 and HeLa monolayer cultures compared with MCF-7 cancer cells. Moreover, ROS generation upon light irradiation was greater in monolayer than in the respective spheroid cultures.

## Conclusions

**Fig 6:**
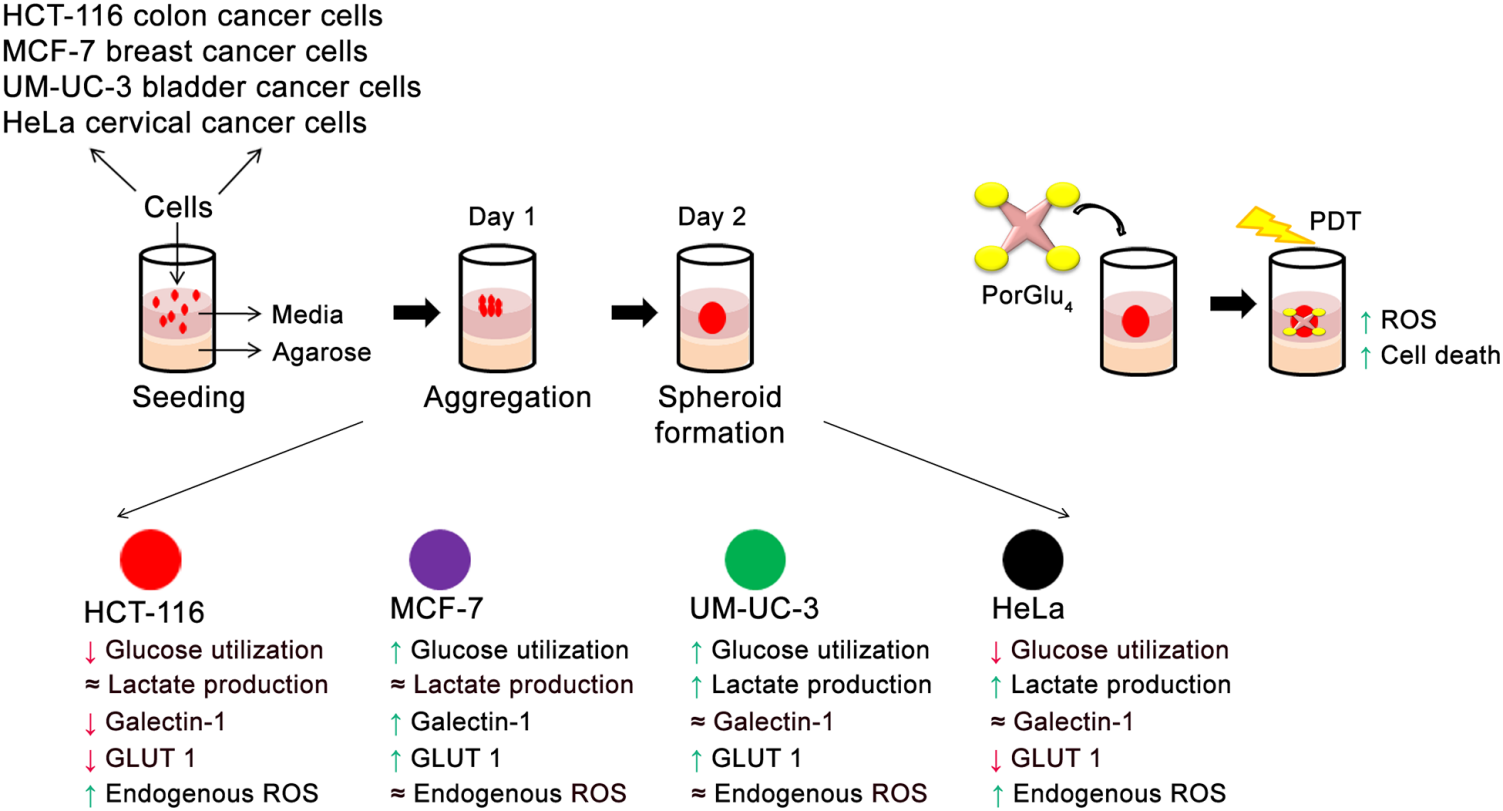
Spheroid formation with HCT-116, MCF-7, UM-UC-3 and HeLa cancer cells; and phototoxicity with PorGlu_4_. The initial aggregation of monolayer cancer cells plated for spheroid formation lasted for 24 h. Within 48–72 h the small clumps were replaced by compact spheroids. The glycolytic status as indicated by glucose usage and lactate production, endogenous levels of ROS, and expression of galectin-1 and GLUT1 are altered during spheroids formation. PorGlu_4_ is non-toxic at these concentrations yet has high toxicity upon PDT. The uptake and phototoxocity of PorGlu_4_ is dependent on the cell line, levels of galectin-1 and GLUT1 in cancer cells and on the *in vitro* (2-D or 3-D) model used.

Spheroids as models to screen therapeutics present distinct advantages [60–63]. The present study provides a detailed characterization of HCT-116, MCF-7, UM-UC-3 and HeLa spheroid cultures with respect to a number of parameters that are critical for screening and designing cancer targeting glyco-PS, including glucose metabolism, oxidative stress during spheroids formation, and related variations in galectin-1 and GLUT1 proteins. This study highlights the metabolic and morphological differences between spheroids and cell cultures that provides a solid framework and specific methods for evaluating the efficacy of glyco-PS and other glycosylated drugs. Overall, for glyco-PS as PDT agents, using spheroids is a more predictive model of tumors than the commonly used monolayer culture (Fig 6).

## Supporting information

S1 Fig Glucose utilization of cancer cells growing in monolayers at 48 h after plating with seeding densities of 5,000 HCT-116 cells per well, 15,000 MCF-7 cells per well, 20,000 UM-UC-3 cells per well and 20,000 HeLa cells per well. The amount of glucose (nmol) was estimated using the fluorescent glucose analog 2NBDG and the results were normalized for mg of protein. Data are means ± S.D. of at least three independent experiments performed in triplicate. ***P< 0.001 compared to glucose utilization in HeLa cells.
(TIF)

S2 Fig Lactate utilization of cancer cells growing in monolayers at 48 h after plating with seeding densities of 5,000 HCT-116 cells per well, 15,000 MCF-7 cells per well, 20,000 UM-UC-3 cells per well and 20,000 HeLa cells per well. The amount of lactate (nmol) was estimated using an enzymatic assay and the results were normalized for mg of protein. Data are means ± S.D. of at least three independent experiments performed in triplicate. ***P< 0.001 compared to glucose utilization in HeLa cells.
(TIF)

S3 Fig Quantitative analysis of galectin-1 (normalized to β-actin) in spheroid cultures expressed as a ratio of the levels found in HeLa spheroids. Data are means ± S.D. of at least five independent experiments. **P< 0.01, ***P< 0.001 compared to galectin-1 protein levels in HeLa spheroids.
(TIF)

S4 Fig Quantitative analysis of GLUT1 (normalized to β-actin) in spheroid cultures expressed as a ratio of the levels found in HeLa spheroids. Data are means ± S.D. of at least five independent experiments. *P< 0.05, ***P< 0.001 compared to GLUT1 protein levels in HeLa spheroids.
(TIF)

S5 Fig Chemical structure of PorGlu_4_. (TIF)

S6 Fig Cytotoxicity of PBS solution containing 0.5% DMSO (v/v) in monolayer cultures at different incubation times (0.5, 1, 2 and 4h). The percentage of cytotoxicity was calculated relatively to control cells (cells incubated with medium). Data are means ± S.D. of at least three independent experiments performed in triplicate.
(TIF)

S7 Fig Non-dark toxicity of PorGlu_4_ at different concentrations (0, 2.25, 4.5 and 9 μM) and uptake time of 4h, in monolayer cultures. The percentage of cytotoxicity was calculated relatively to control cells (untreated cells). Data are means ± S.D. of at least three independent experiments performed in triplicate.
(TIF)

S8 Fig Intracellular uptake of PorGlu_4_ by HCT-116, MCF-7, UM-UC-3 and HeLamonolayer cultures. The concentration of PorGlu_4_ was determined by fluorescence spectroscopy (λ_excitation_ at 410 nm and λ_emission_ at 702 nm) after incubation of cancer cells with 0, 2.25, 4.5 or 9 μM of PorGlu_4_ for 0.5, 1, 2 or 4 h and the results normalized to protein quantity. Data are means ± S.D. of at least three independent experiments performed in triplicate.
(TIF)

S9 Fig Intracellular uptake of PorGlu_4_ by HCT-116, MCF-7, UM-UC-3 and HeLa growing in monolayer or spheroid cultures. The concentration of PorGlu_4_ was determined by fluorescence spectroscopy after incubation of cancer cells with 9 μM PorGlu_4_ during 4 h (λ_excitation_ at 410 nm and λ_emission_ at 702 nm) and the results normalized to protein quantity. Data are means ± S.D. of at least three independent experiments performed in triplicate. **P< 0.01, ***P< 0.001 compared to PorGlu_4_ uptake by the respective cancer cell line growing in monolayers.
(TIF)

S10 Fig Non-dark toxicity of PorGlu_4_ at 9 μM and uptake time of 4 h in spheroid cultures as determined using the LDH assay. The percentage of cytotoxicity was calculated relatively to control cells (untreated cells). Data are means ± S.D. of at least three independent experiments performed in triplicate.
(TIF)

S11 Fig Cytotoxicity at 24 h after PDT with PorGlu_4_ in cancer cells growing in monolayers, determined using the MTT assay. Data are means ± S.D. of at least three independent experiments performed in triplicate. **P< 0.01, ***P< 0.001 compared to MTT reduction (%) of control cells (untreated cells).
(TIF)

S12 Fig Cytotoxicity at 24 h after PDT with PorGlu_4_ in cancer cells growing in spheroids, determined using the LDH assay. Data are means ± S.D. of at least three independent experiments performed in triplicate. *P< 0.05, **P< 0.01, ***P< 0.001 compared to LDH reduction (%) of control cells (untreated cells).
(TIF)

S13 Fig Quantification of DCF fluorescence increase as a measure of endogenous ROS in cancer cells growing as monolayers or spheroids. ROS levels are expressed as a ratio of the levels found on the respective cell line growing in monolayers. Data are means ± S.D. of at least three independent experiments performed in triplicate. **P< 0.01, ***P< 0.001 compared to DCF fluorescence in the respective cell line growing in monolayers.
(TIF)

S14 Fig Quantification of DCF fluorescence increase as a measure of ROS production after PDT in cancer cells growing in monolayers, after PDT with PorGlu_4_. ROS levels are expressed as a ratio of the levels found on the respective control cells and normalized to mg of protein. Data are means ± S.D. of at least three independent experiments performed in triplicate. **P< 0.01, ***P< 0.001 compared to ROS production after PDT in control cells. ^$^*P*< 0.05, ^$ $ $^*P*< 0.001 compared to ROS production after PDT in MCF-7 cancer cells.
(TIF)

S15 Fig Quantification of DCF fluorescence increase (as a measure of ROS production after PDT) in cancer cells growing in spheroids, after PDT with PorGlu_4_. ROS levels are expressed as a ratio of the levels found on the respective control cells and normalized to mg of protein. Data are means ± S.D. of at least three independent experiments performed in triplicate. ***P< 0.001 compared to ROS production after PDT in control cells.
(TIF)

S1 Table HCT-116 spheroid size and cellular characteristics as a function of age (24, 48 and 72h) and number of platted cells (2,500, 5,000, 10,000 and 20,000 cells per well). (TIF)

S2 Table MCF-7 spheroid size and cellular characteristics as a function of age (24, 48 and 72h) and number of platted cells (5,000, 10,000, 15,000 and 20,000 cells per well). (TIF)

S3 Table UM-UC-3 spheroid size and cellular characteristics as a function of age (24, 48 and 72h) and number of platted cells (2,500, 5,000, 10,000 and 20,000 cells per well). (TIF)

S4 Table HeLa spheroid size and cellular characteristics as a function of age (24, 48 and 72h) and number of platted cells (2,500, 5,000, 10,000 and 20,000 cells per well). (TIF)

## Abbreviations

DMEM: Dulbecco´s Modified Eagle´s Medium
DMSO: Dimethylsulfoxide
EMEM: Eagle´s Minimum Essential Medium
GLUT1: Glucose transporter 1
H_2_DCFDA: 2′,7′ -dichloro dihydrofluorescein diacetate
LDH: Lactate dehydrogenase
MTT: 3-[4,5-dimethylthiazol-2-yl]-2,5-diphenyl-tetrazolium bromide
PBS: Phosphate buffered saline
PDT: Photodynamic therapy
Por: Porphyrin
PorGlu_4_: 5,10,15,20-tetrakis-(4–1’-thio-glucosyl-2,3,5,6-tetrafluorophenyl)-porphyrin
PS: Photosensitizer
ROS: Reactive oxygen species
SDS: Sodium dodecyl sulfate
