# Supplementary figures and images for: Cancer cell spheroids are a better screen for the photodynamic efficiency of glycosylated photosensitizers
Source: PLoS One. 2017 May 17;12(5):e0177737. doi: 10.1371/journal.pone.0177737 (PMC5435229; doi:10.1371/journal.pone.0177737)

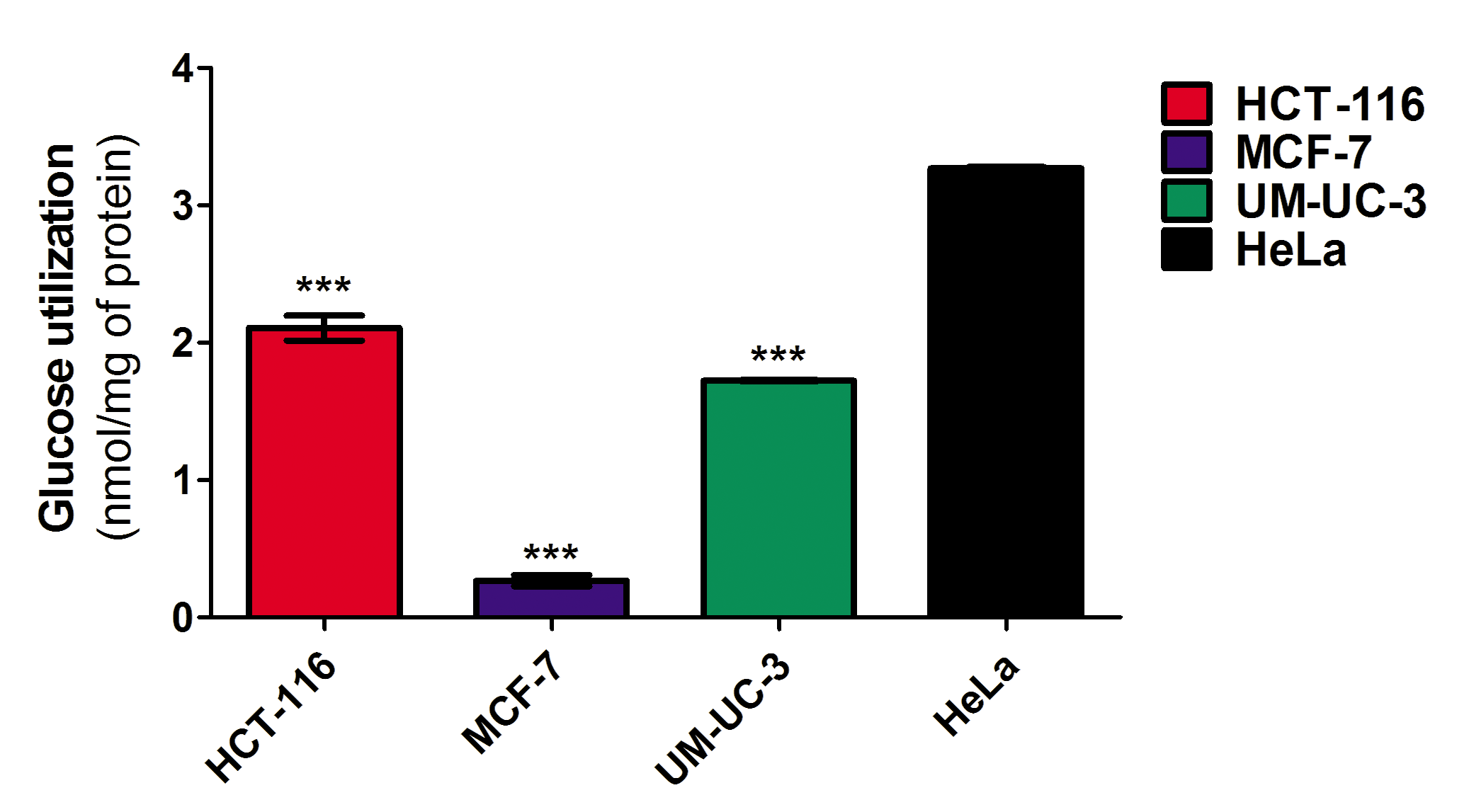

Supplement: S1 Fig — The amount of glucose (nmol) was estimated using the fluorescent glucose analog 2NBDG and the results were normalized for mg of protein. Data are means ± S.D. of at least three independent experiments performed in triplicate. ***P< 0.001 compared to glucose utilization in HeLa cells. (TIF) [file pone.0177737.s001.tif]

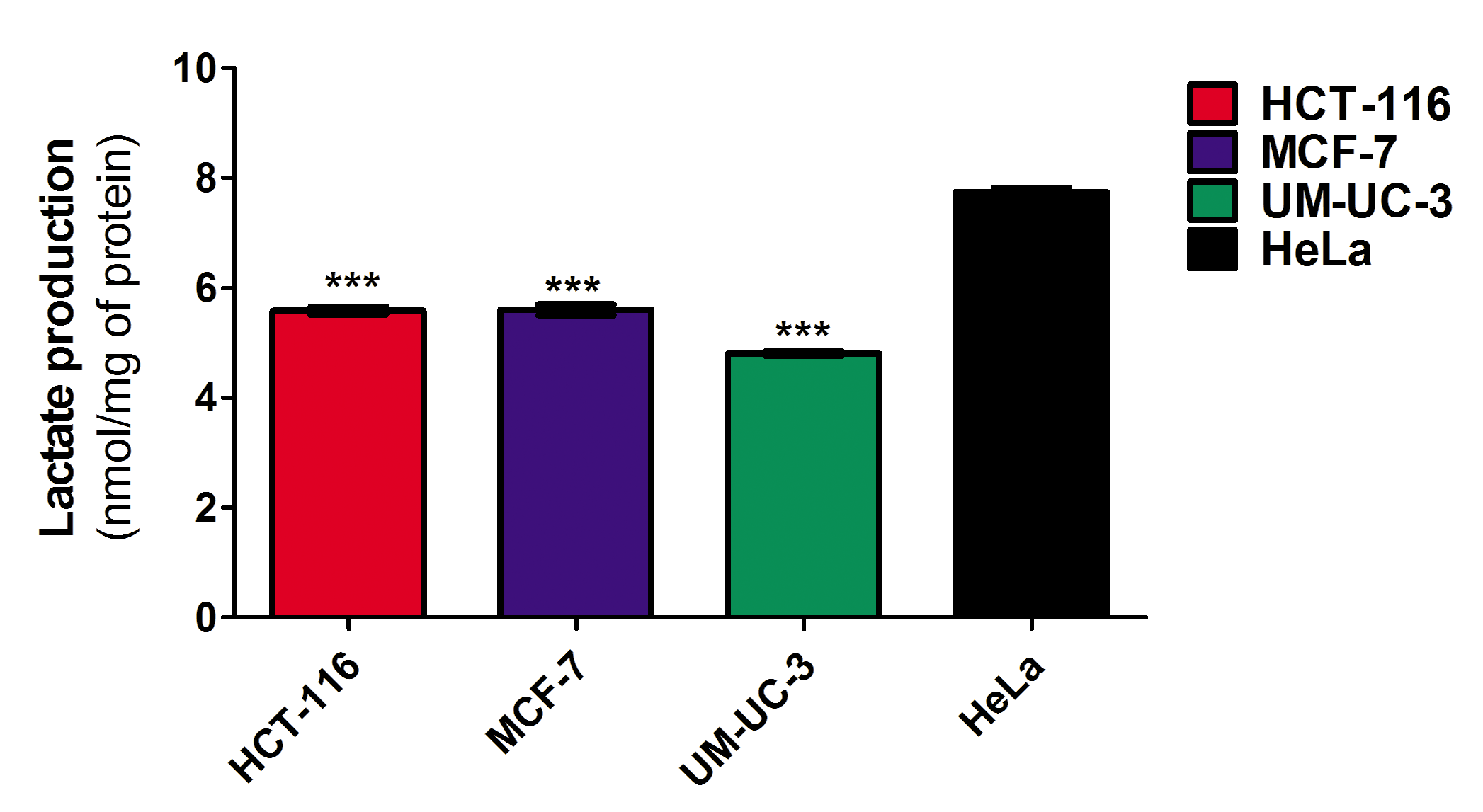

Supplement: S2 Fig — The amount of lactate (nmol) was estimated using an enzymatic assay and the results were normalized for mg of protein. Data are means ± S.D. of at least three independent experiments performed in triplicate. ***P< 0.001 compared to glucose utilization in HeLa cells. (TIF) [file pone.0177737.s002.tif]

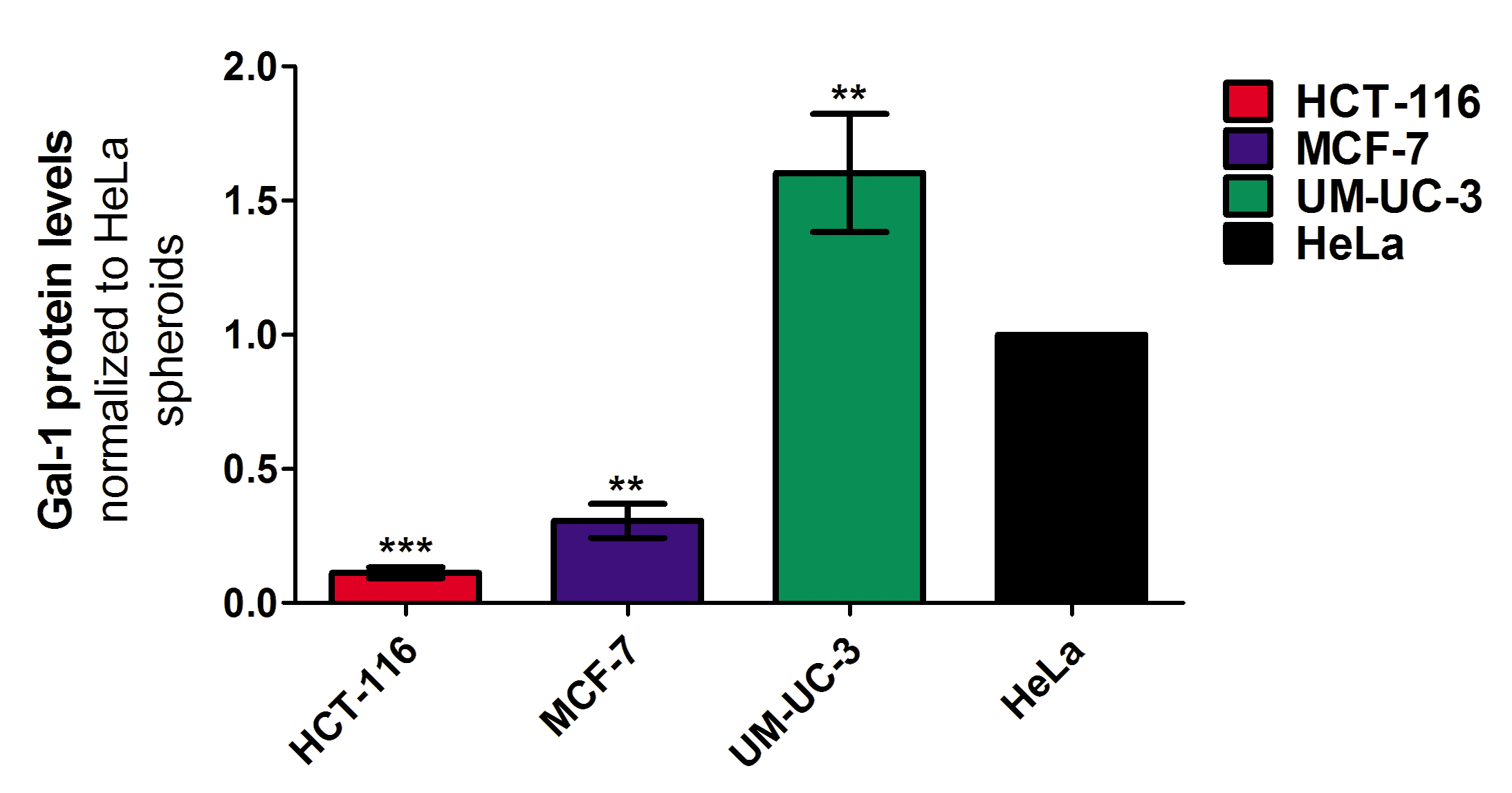

Supplement: S3 Fig — Data are means ± S.D. of at least five independent experiments. **P< 0.01, ***P< 0.001 compared to galectin-1 protein levels in HeLa spheroids. (TIF) [file pone.0177737.s003.tif]

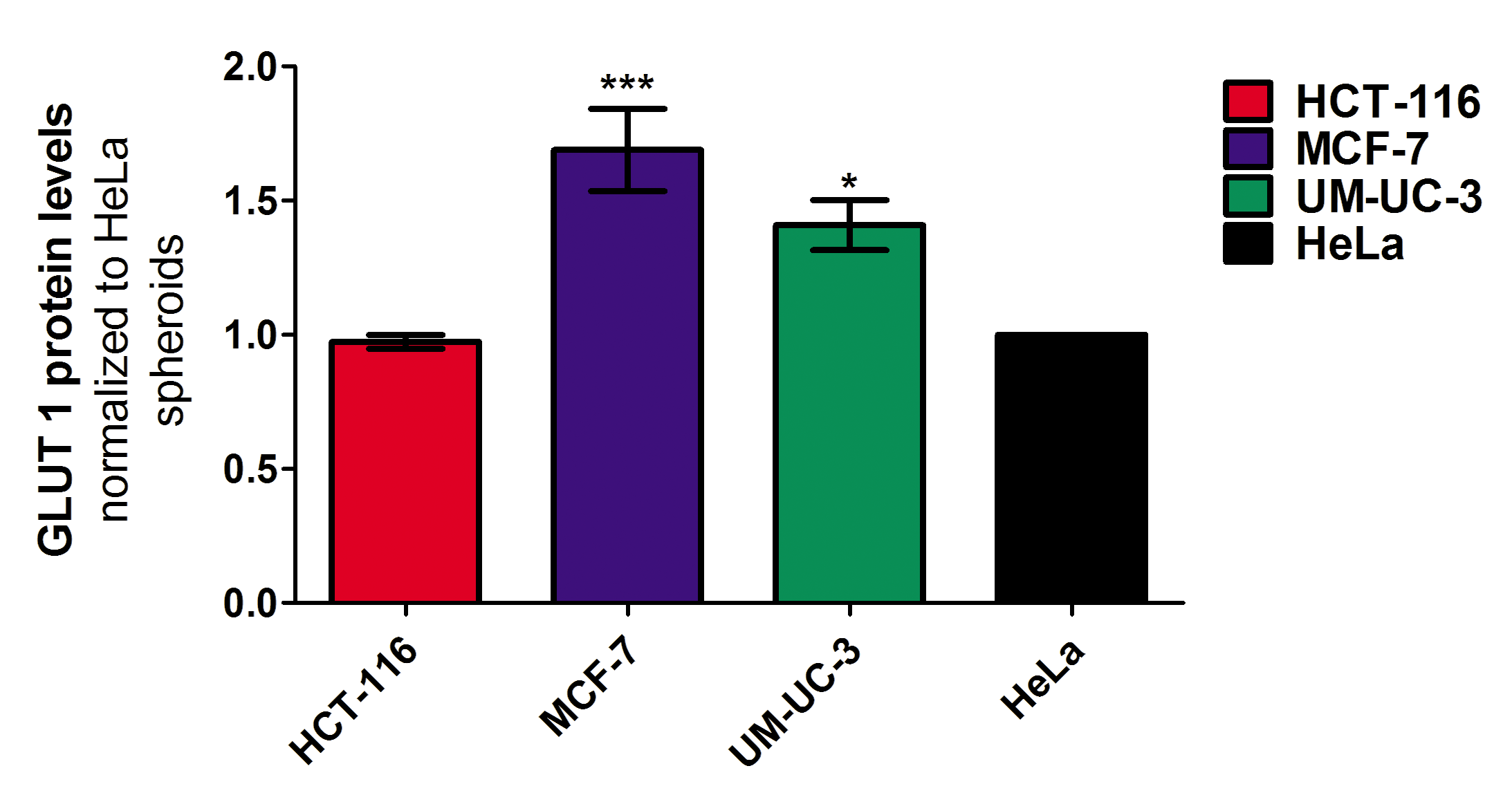

Supplement: S4 Fig — Data are means ± S.D. of at least five independent experiments. *P< 0.05, ***P< 0.001 compared to GLUT1 protein levels in HeLa spheroids. (TIF) [file pone.0177737.s004.tif]

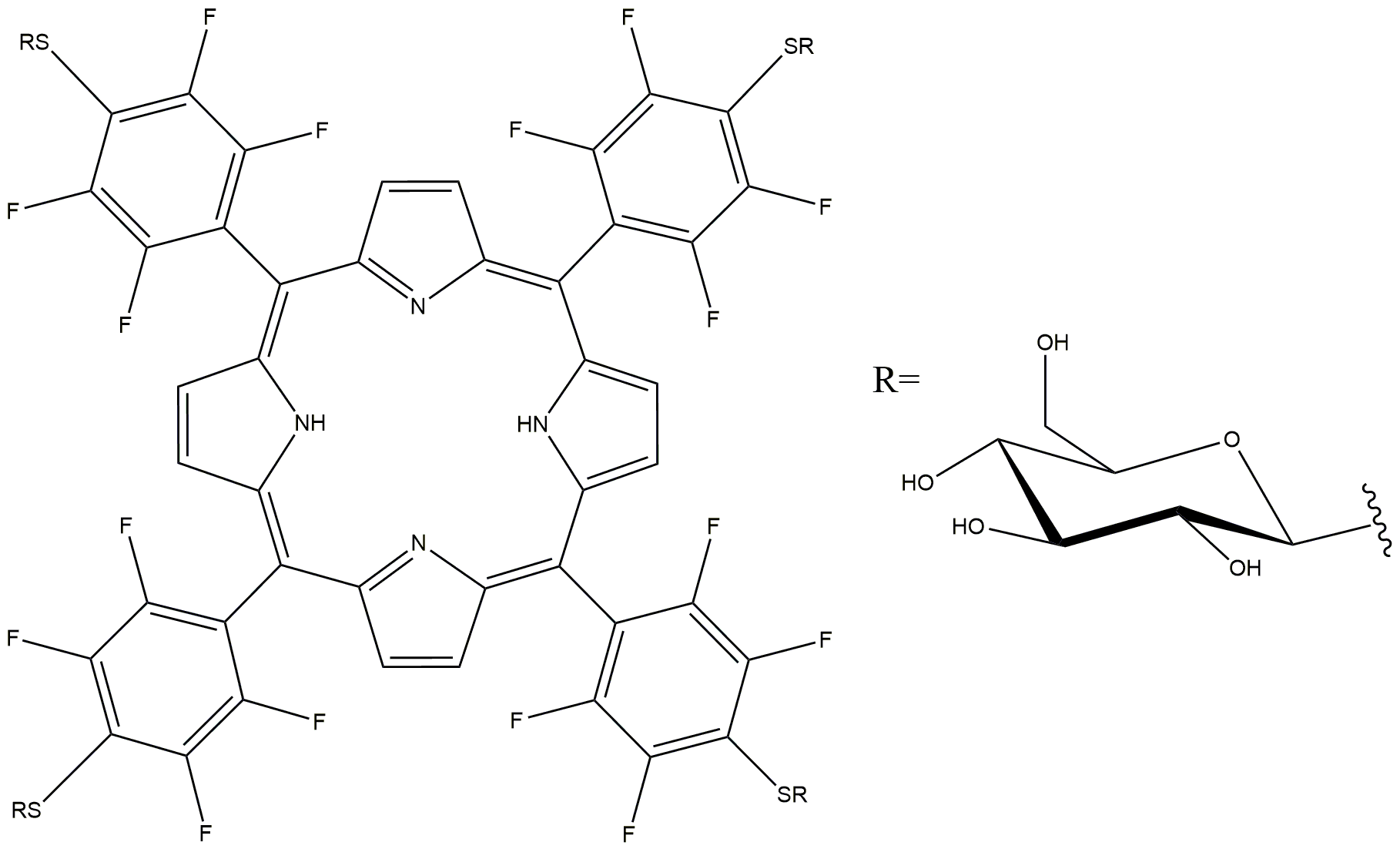

Supplement: S5 Fig — (TIF) [file pone.0177737.s005.tif]

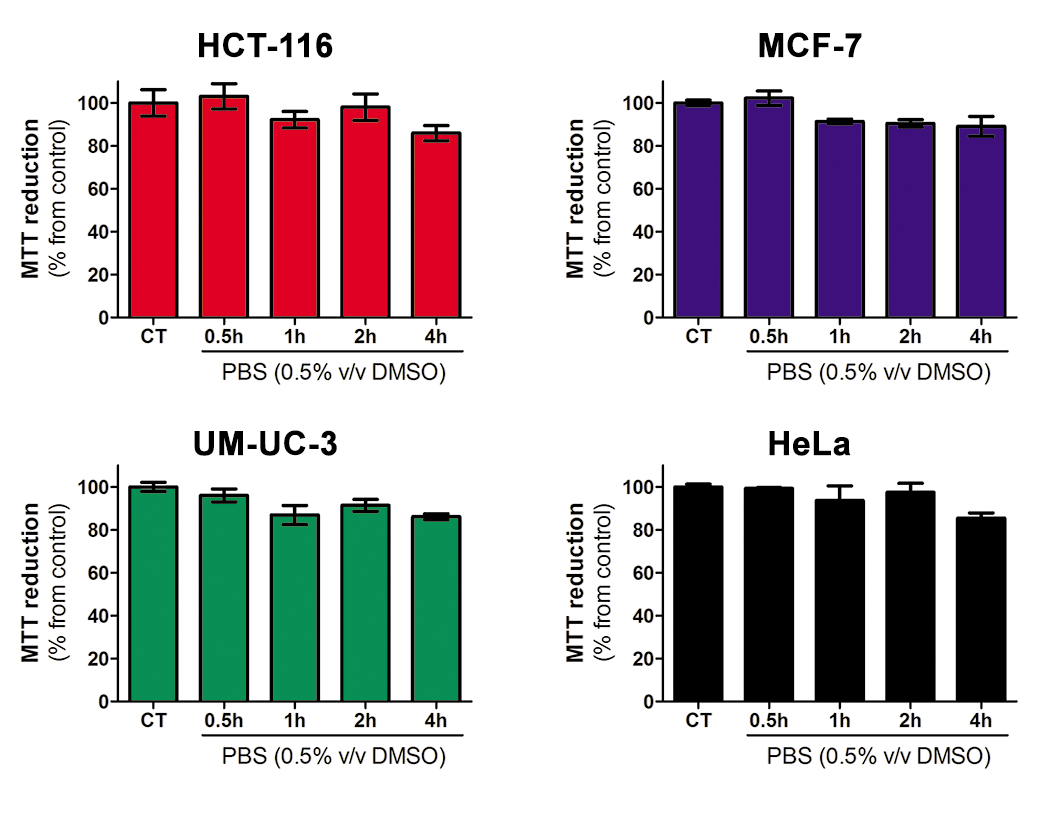

Supplement: S6 Fig — The percentage of cytotoxicity was calculated relatively to control cells (cells incubated with medium). Data are means ± S.D. of at least three independent experiments performed in triplicate. (TIF) [file pone.0177737.s006.tif]

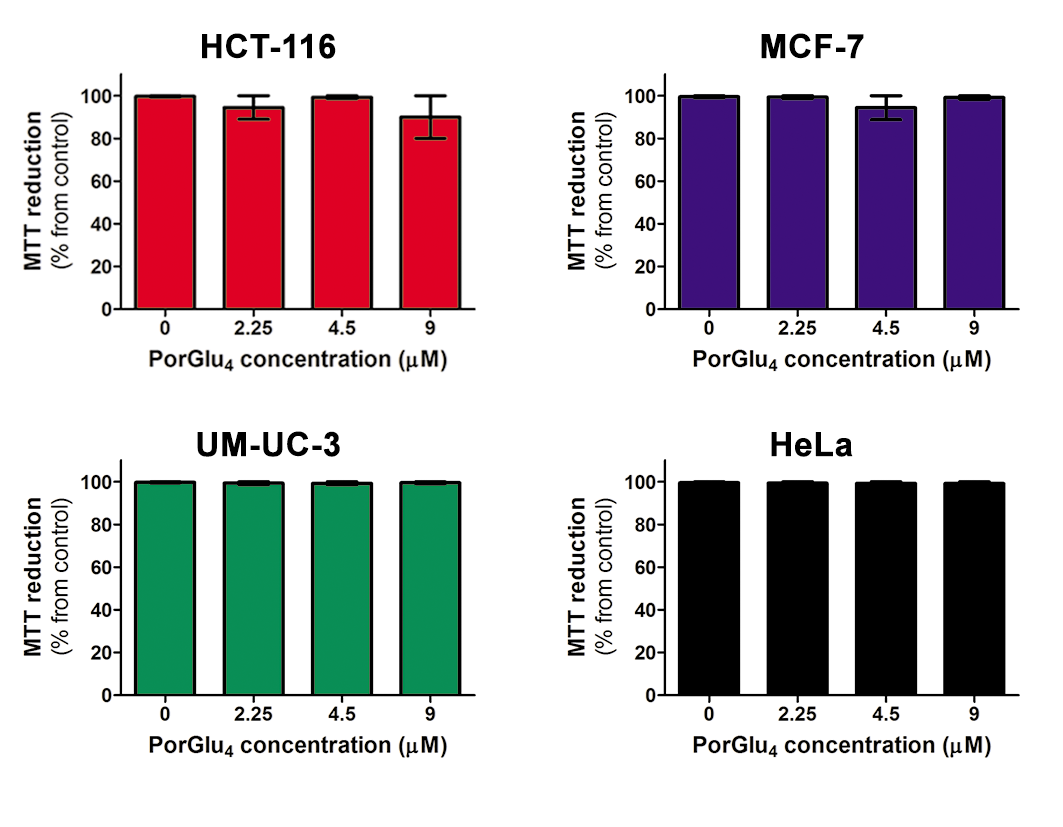

Supplement: S7 Fig — The percentage of cytotoxicity was calculated relatively to control cells (untreated cells). Data are means ± S.D. of at least three independent experiments performed in triplicate. (TIF) [file pone.0177737.s007.tif]

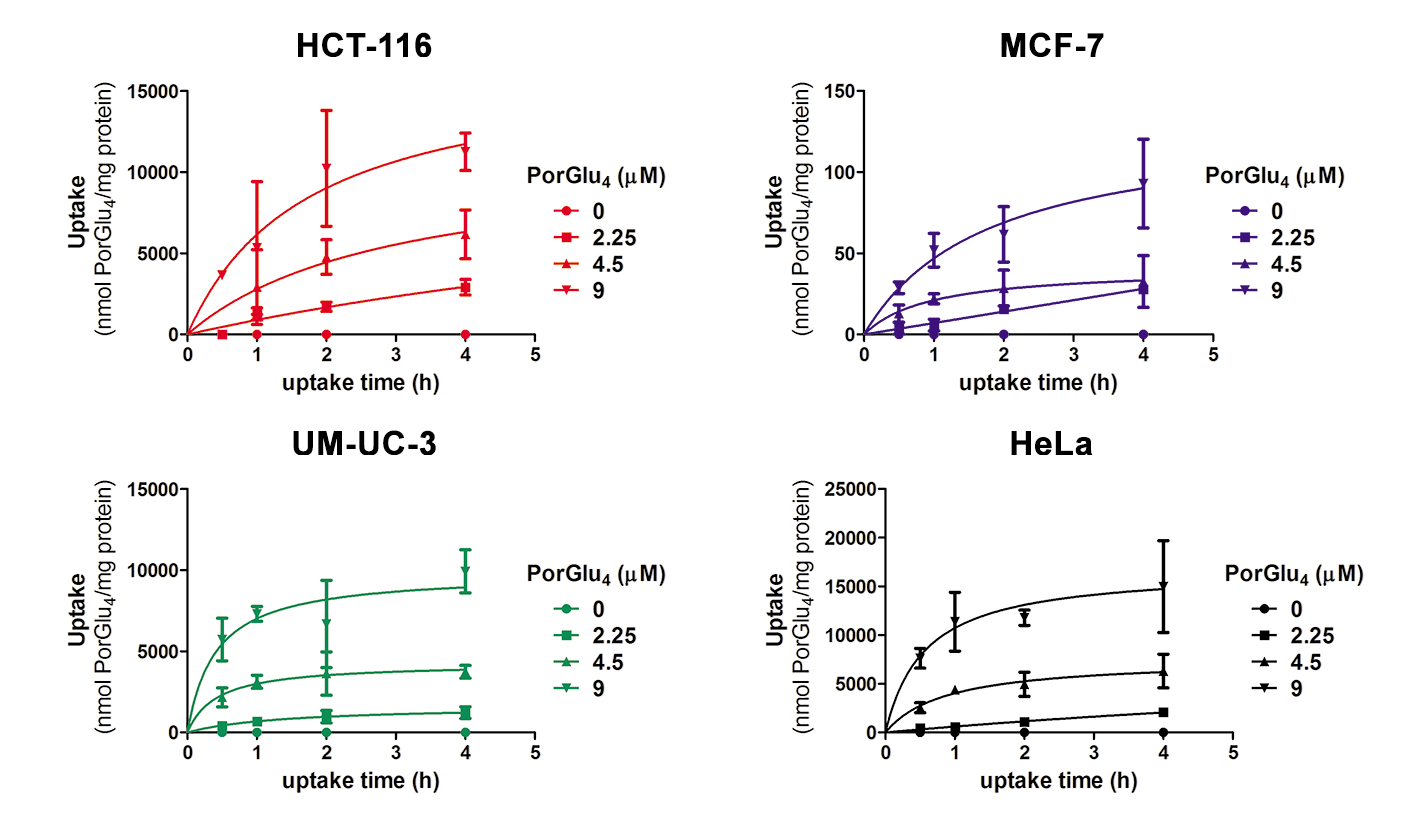

Supplement: S8 Fig — The concentration of PorGlu4 was determined by fluorescence spectroscopy (λexcitation at 410 nm and λemission at 702 nm) after incubation of cancer cells with 0, 2.25, 4.5 or 9 μM of PorGlu4 for 0.5, 1, 2 or 4 h and the results normalized to protein quantity. Data are means ± S.D. of at least three independent experiments performed in triplicate. (TIF) [file pone.0177737.s008.tif]

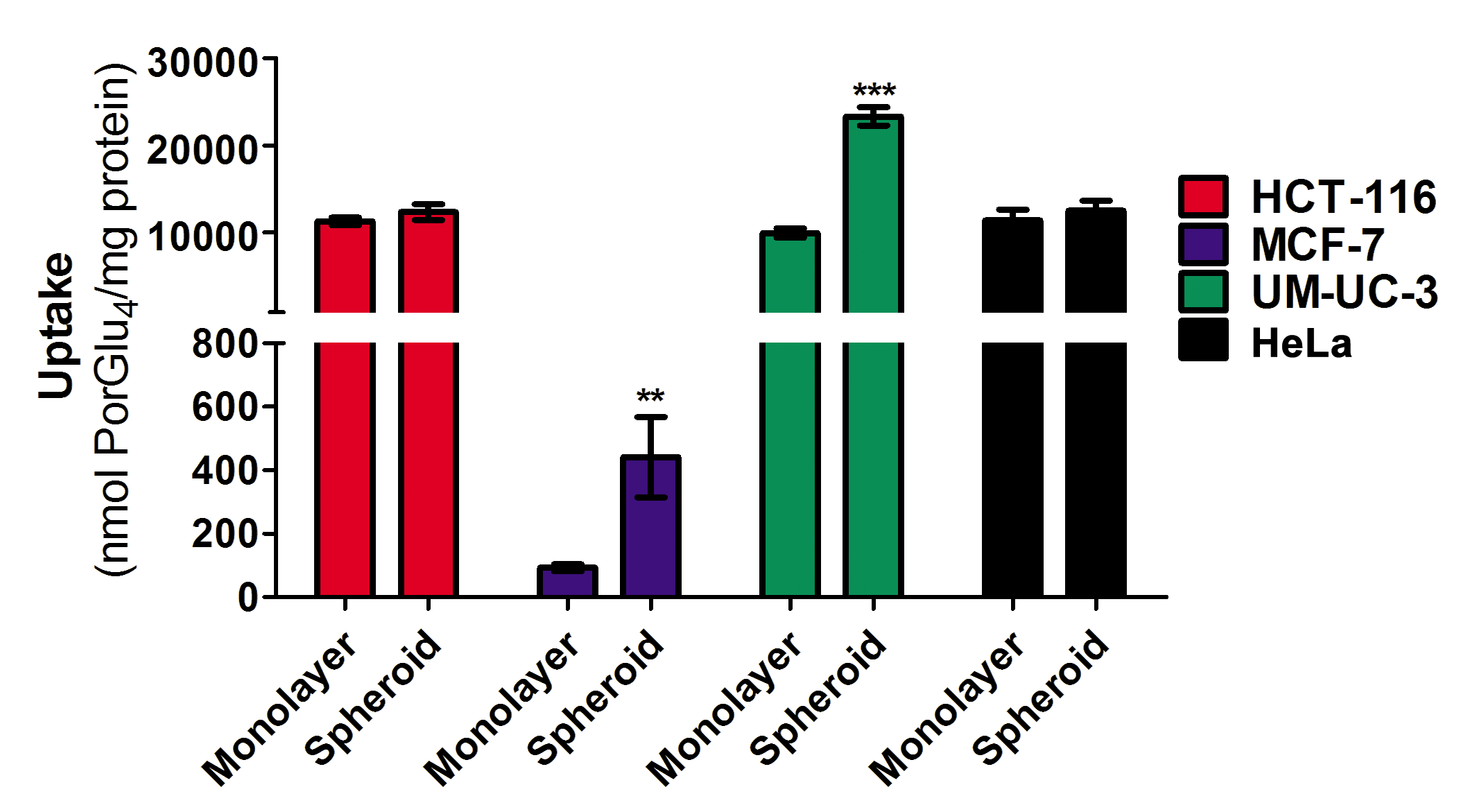

Supplement: S9 Fig — The concentration of PorGlu4 was determined by fluorescence spectroscopy after incubation of cancer cells with 9 μM PorGlu4 during 4 h (λexcitation at 410 nm and λemission at 702 nm) and the results normalized to protein quantity. Data are means ± S.D. of at least three independent experiments performed in triplicate. **P< 0.01, ***P< 0.001 compared to PorGlu4 uptake by the respective cancer cell line growing in monolayers. (TIF) [file pone.0177737.s009.tif]

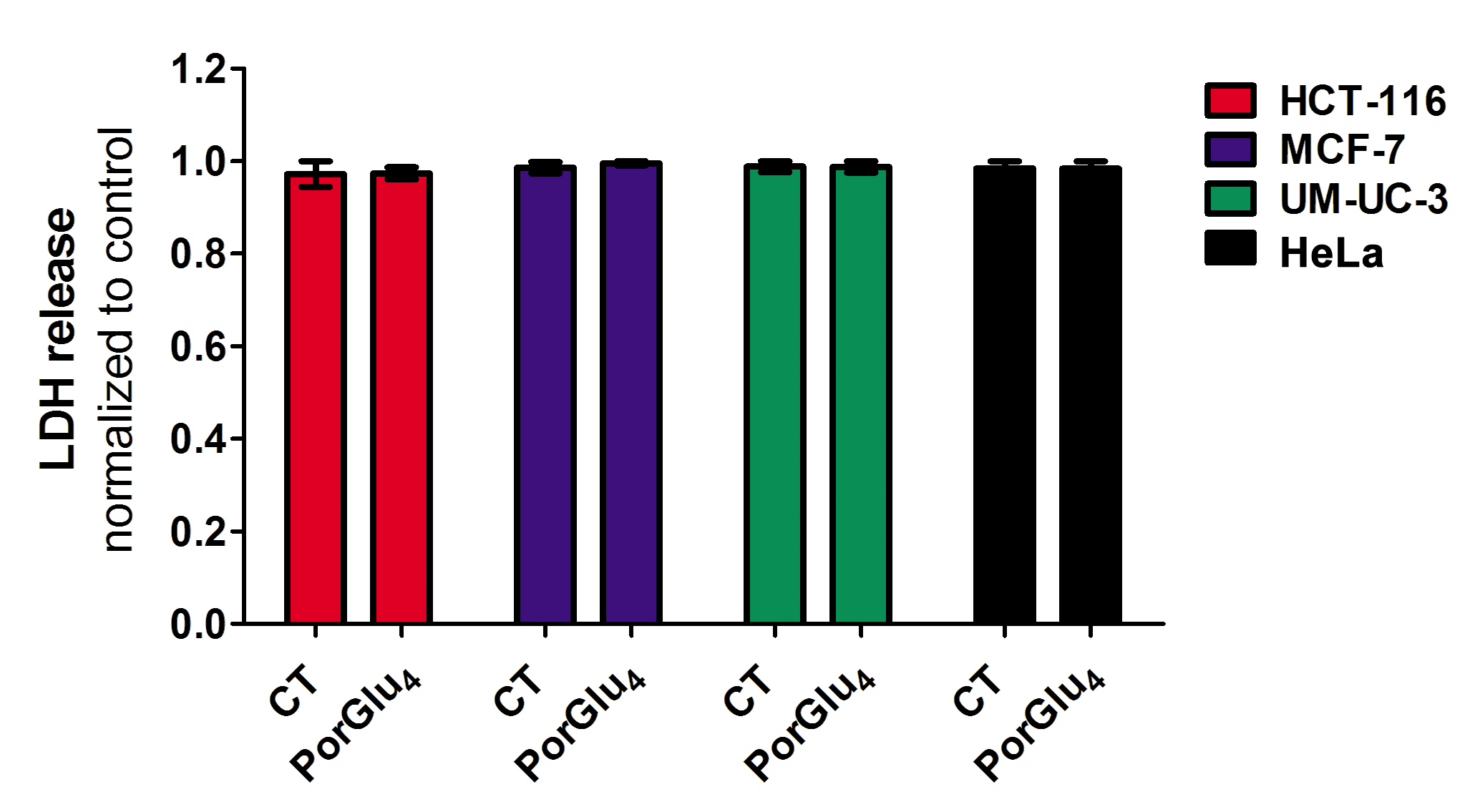

Supplement: S10 Fig — The percentage of cytotoxicity was calculated relatively to control cells (untreated cells). Data are means ± S.D. of at least three independent experiments performed in triplicate. (TIF) [file pone.0177737.s010.tif]

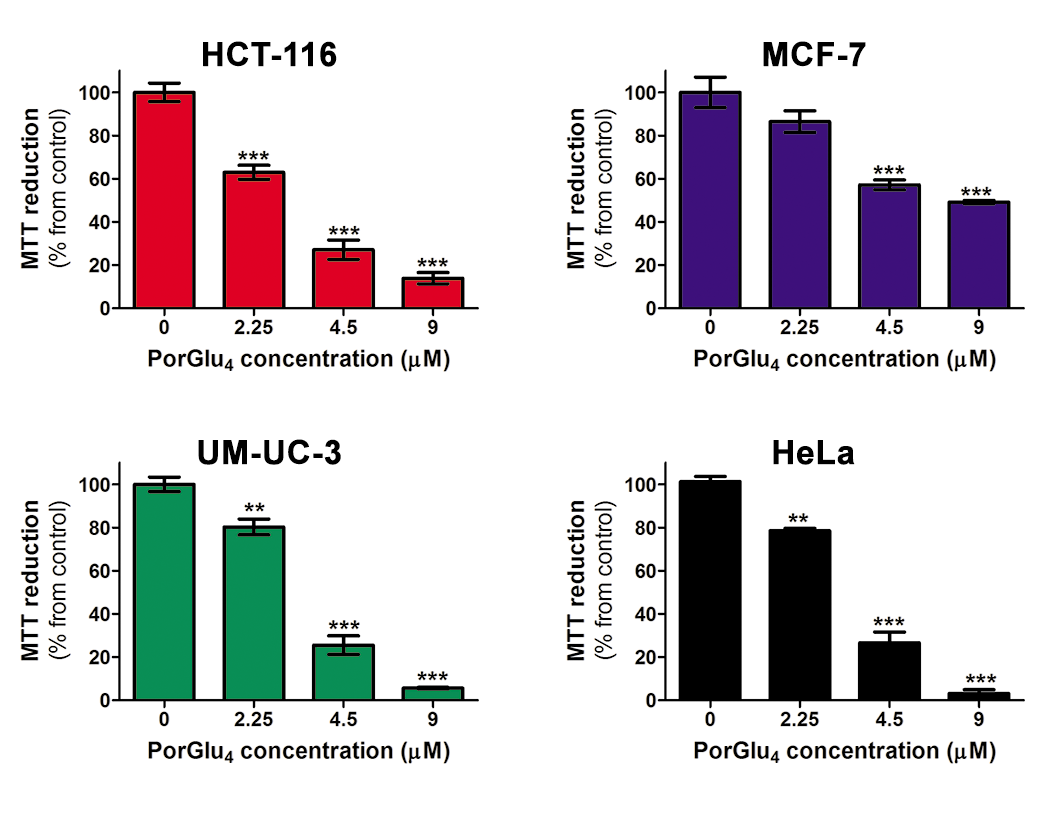

Supplement: S11 Fig — Data are means ± S.D. of at least three independent experiments performed in triplicate. **P< 0.01, ***P< 0.001 compared to MTT reduction (%) of control cells (untreated cells). (TIF) [file pone.0177737.s011.tif]

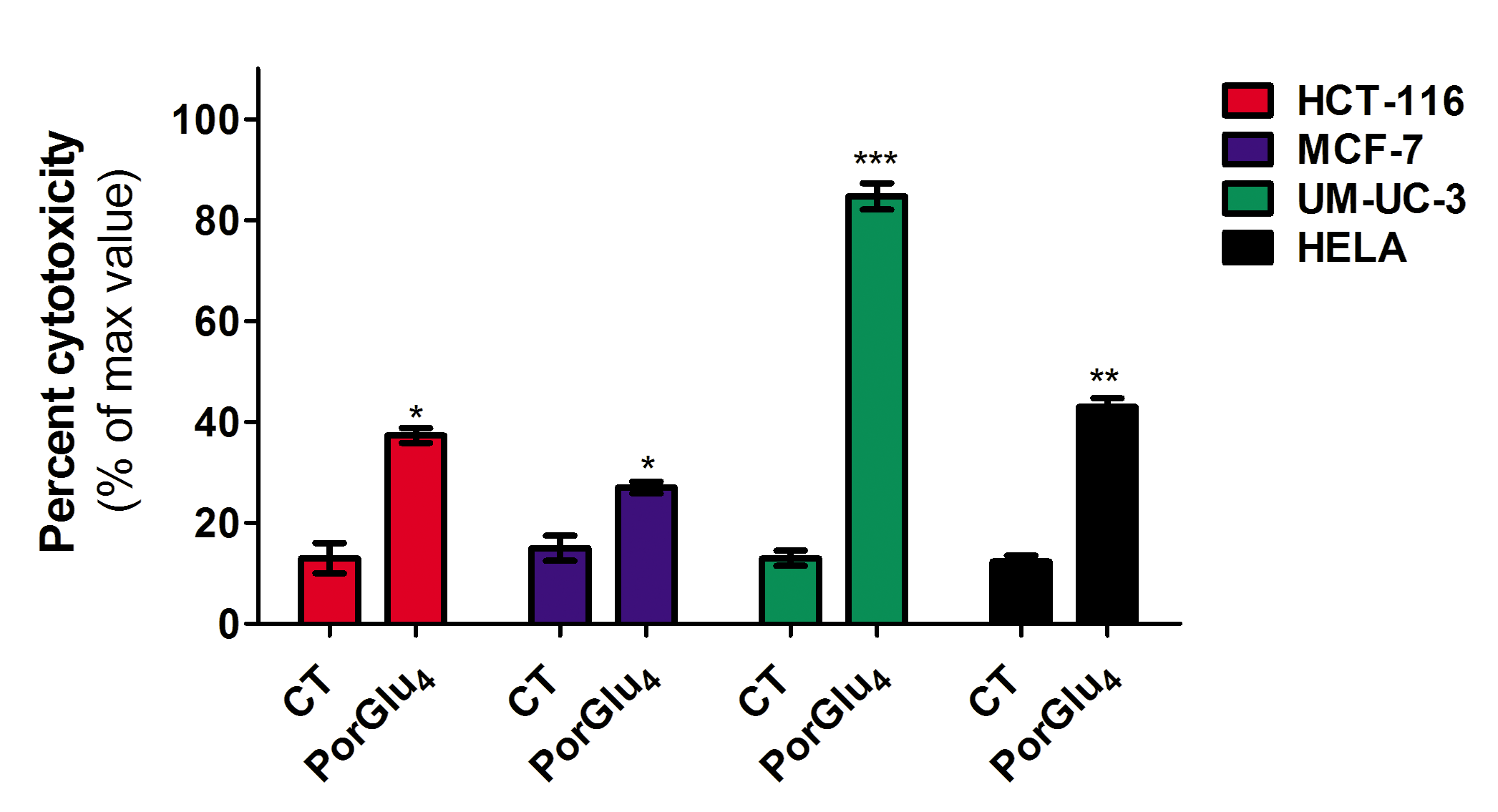

Supplement: S12 Fig — Data are means ± S.D. of at least three independent experiments performed in triplicate. *P< 0.05, **P< 0.01, ***P< 0.001 compared to LDH reduction (%) of control cells (untreated cells). (TIF) [file pone.0177737.s012.tif]

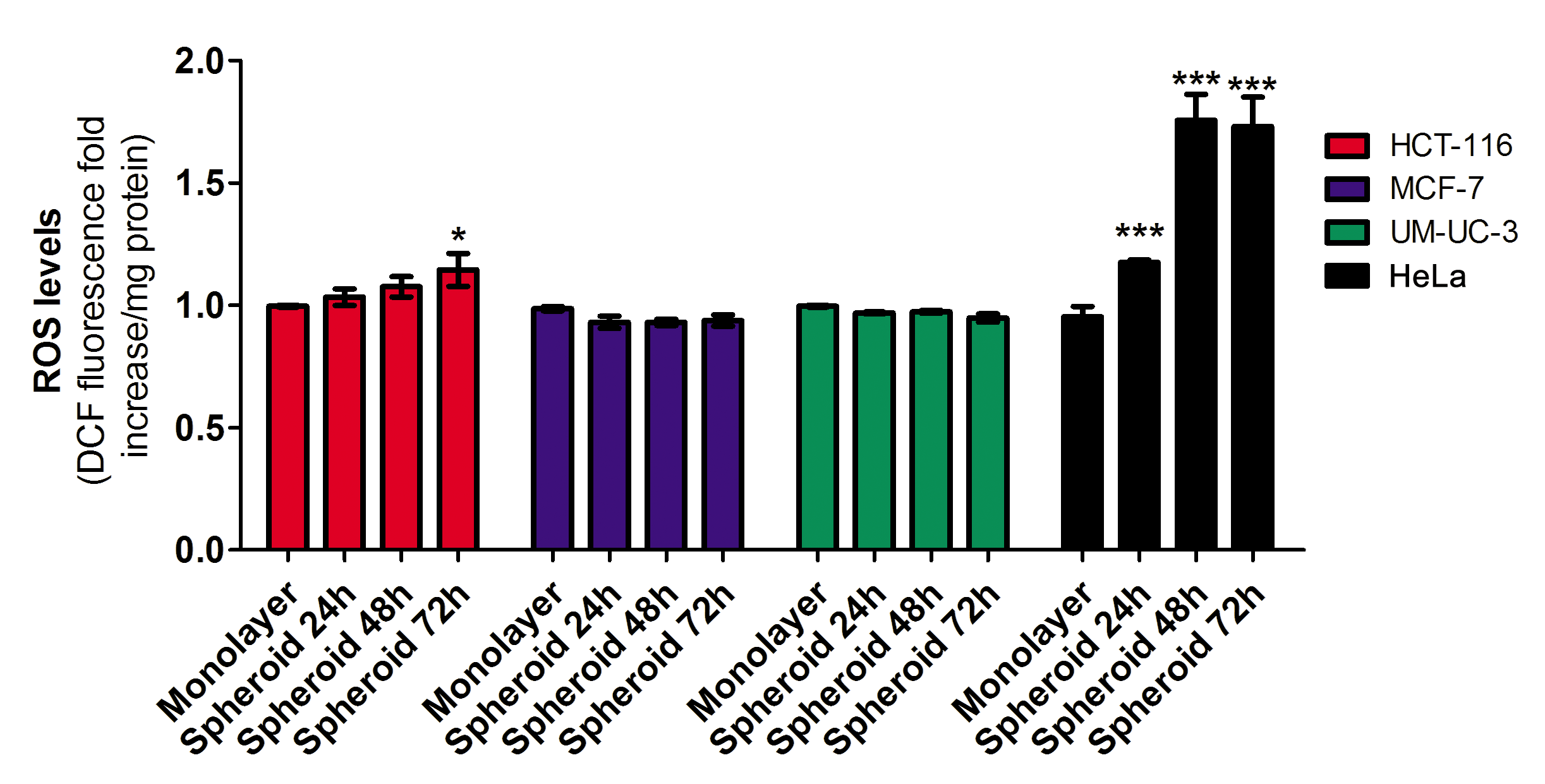

Supplement: S13 Fig — ROS levels are expressed as a ratio of the levels found on the respective cell line growing in monolayers. Data are means ± S.D. of at least three independent experiments performed in triplicate. **P< 0.01, ***P< 0.001 compared to DCF fluorescence in the respective cell line growing in monolayers. (TIF) [file pone.0177737.s013.tif]

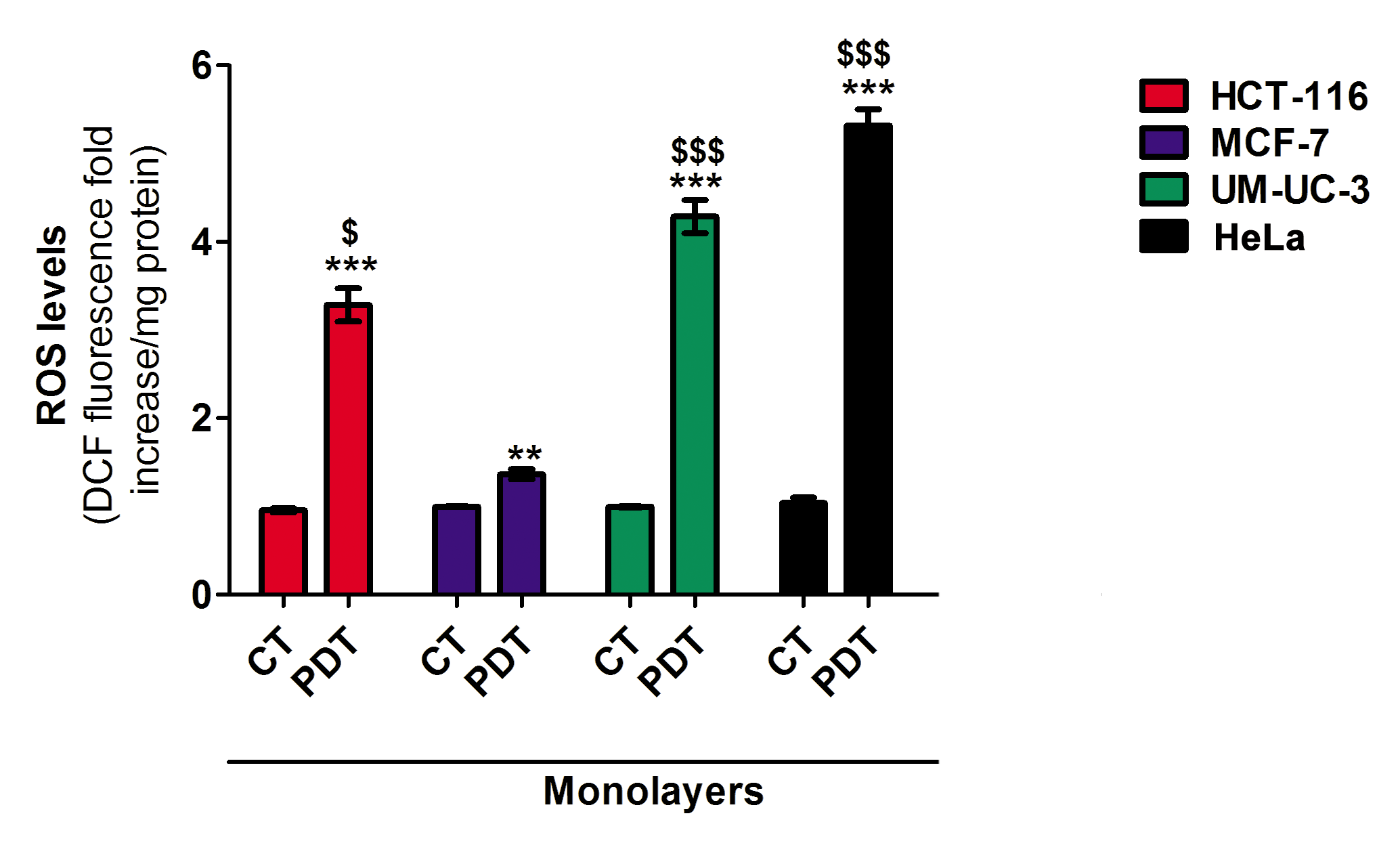

Supplement: S14 Fig — ROS levels are expressed as a ratio of the levels found on the respective control cells and normalized to mg of protein. Data are means ± S.D. of at least three independent experiments performed in triplicate. **P< 0.01, ***P< 0.001 compared to ROS production after PDT in control cells. $P< 0.05, $ $ $P< 0.001 compared to ROS production after PDT in MCF-7 cancer cells. (TIF) [file pone.0177737.s014.tif]

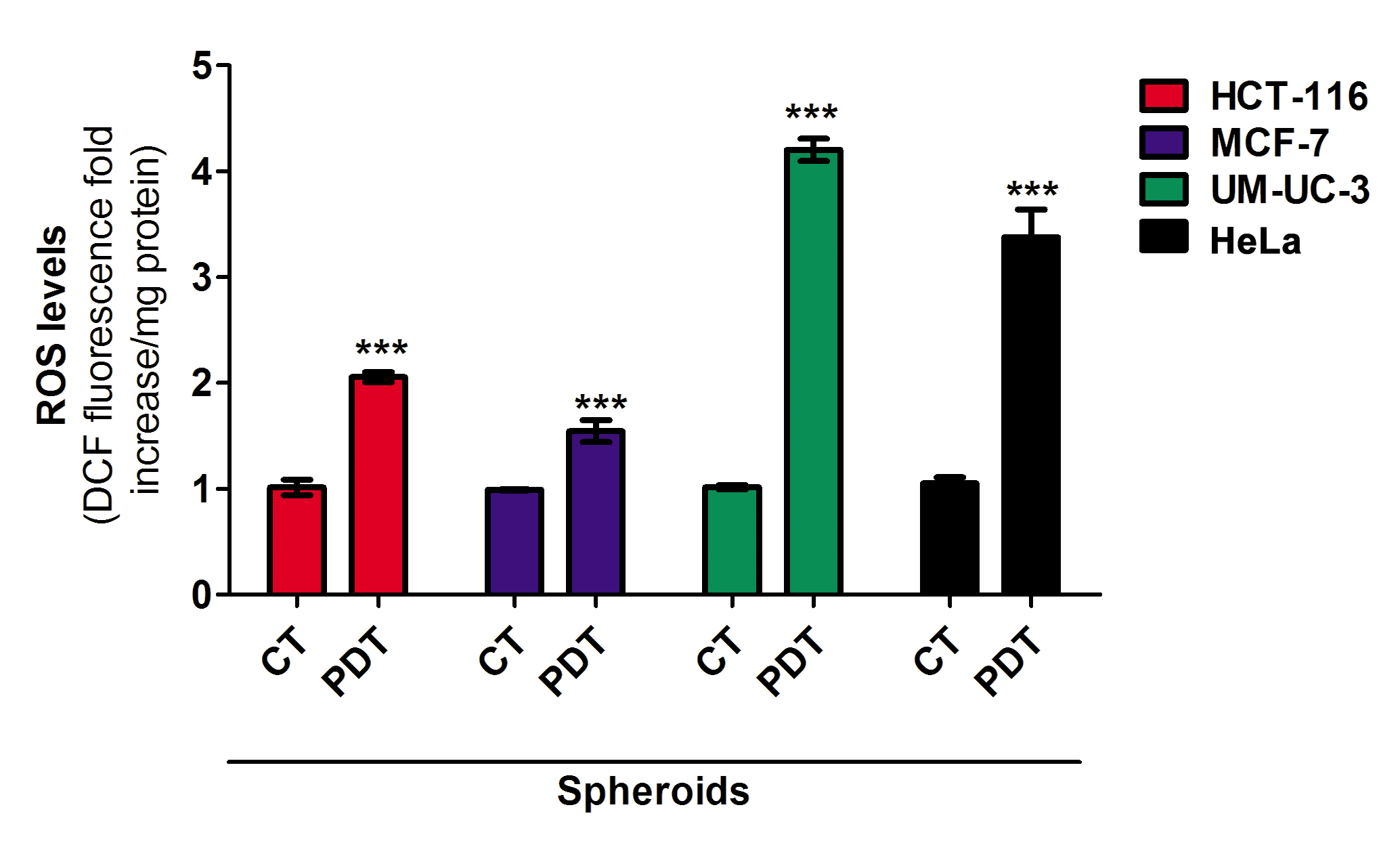

Supplement: S15 Fig — ROS levels are expressed as a ratio of the levels found on the respective control cells and normalized to mg of protein. Data are means ± S.D. of at least three independent experiments performed in triplicate. ***P< 0.001 compared to ROS production after PDT in control cells. (TIF) [file pone.0177737.s015.tif]

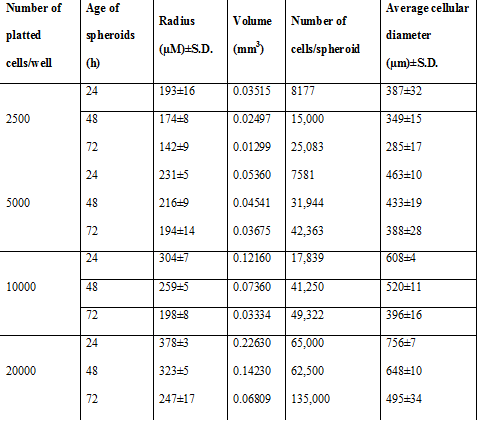

Supplement: S1 Table — (TIF) [file pone.0177737.s016.tif]

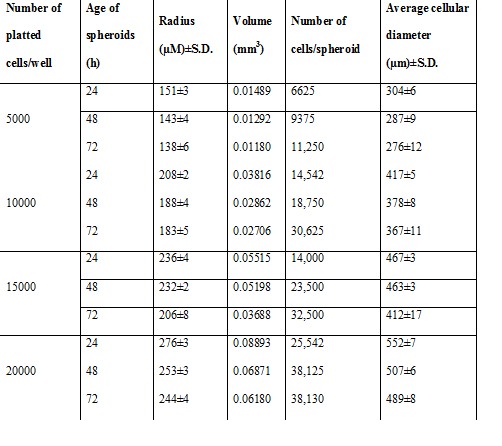

Supplement: S2 Table — (TIF) [file pone.0177737.s017.tif]

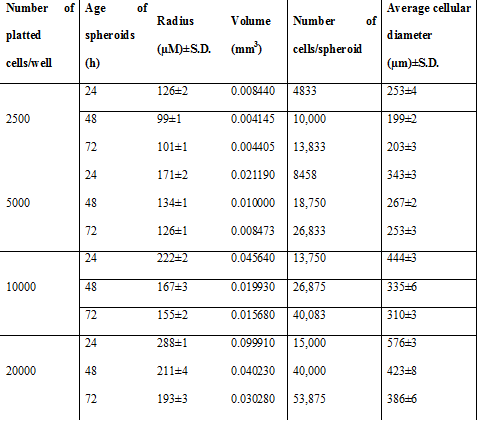

Supplement: S3 Table — (TIF) [file pone.0177737.s018.tif]

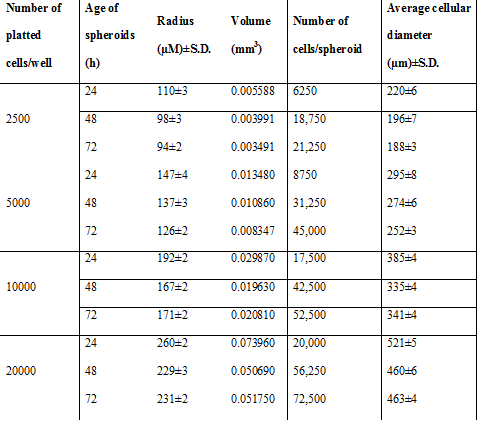

Supplement: S4 Table — (TIF) [file pone.0177737.s019.tif]
